# Anoctamin 5 mutation leads to abnormal bone homeostasis of GDD by regulating AMPK-dependent glucose metabolism

**DOI:** 10.3389/fendo.2026.1703491

**Published:** 2026-02-04

**Authors:** Hongyu Li, Huichong Xu, Kaiwen Sun, Shengnan Wang, Mingyue Zhang, Liqi Lin, Shuai Zhang, Rui Dong, Ying Hu

**Affiliations:** Beijing Institute of Dental Research, Beijing Stomatological Hospital, Capital Medical University, Beijing, China

**Keywords:** AMPK, ANO5, GDD, glucose metabolism, osteoclastogenesis, osteogenesis

## Abstract

**Introduction:**

Gnathodiaphyseal Dysplasia (GDD) characterized by enhanced bone mass and spontaneous fractures is a rare autosomal dominant genetic disease caused by *Anoctamin 5 (ANO5)* mutations. Deletion or mutation in ANO5 exhibited abnormal bone metabolism of GDD manifested by increased osteogenesis and reduced osteoclastogenesis. However, the mechanism is not fully understood.

**Methods:**

*Ano5^Cys360Ty^*^r^ knock-in mouse model was used. Mouse calvarial osteoblast (mCOB) and bone marrow macrophage (BMM) from homozygous knock-in (Ano5KI/KI) and wildtype mice were cultured in vitro. Compound C was selected to inhibit AMPK activity.

**Results:**

We found that *Ano5^Cys360Ty^*^r^ mutation accelerated glycolysis and PGC1a-dependent mitochondrial respiration of osteoblast. Abnormal mitochondrial structure and function caused by PGC1b downregulation was observed in *Ano5*^KI/KI^ osteoclast. Furthermore, ANO5 mutation promoted the phosphorylation of AMPK, the classical sensor of energy metabolism. Inhibiting AMPK reduced glycolysis in osteoblast and maintained the mitochondrial homeostasis between osteoblast and osteoclast by suppressing PGC1a and promoting PGC1b respectively, to restrain bone formation and restore osteoclastogenesis. AMPK inhibitor reversed the bone phenotype of GDD by restraining bone formation and restoring osteoclastogenesis.

**Discussion:**

We highlighted the critical role of glucose metabolic distemperedness mediated by AMPK activation in GDD, which developing a potential therapeutic strategy targeted for treatment of GDD.

## 1 Introduction

GDD is a rare autosomal dominant genetic disease involved in deformity of the jaw and tubular bone. Patients with GDD suffer from mandibular fibrous lesions, maxillofacial swell, abnormal eruption teeth eruption, and osteomyelitis. Frequent fractures of long bone are the most common clinical phenotype of GDD and imaging examination shows cortical thickening accompanied by tibial curvature (1, 2). The pathogenic gene is mapped to *anoctamin 5 (ANO5; GDD1; TMEM16E)* gene located in chromosome 11q14.3-15.1 region, which encodes a 913-amino-acid protein containing ten transmembrane domains (2, 3). ANO5 displays plasma membrane localization and a role in phospholipid scrambling, chlorion transport, and calcium homeostasis (4–6). Heretofore, 31 GDD families and sporadic patients have been reported, for which 10 missense mutations and 1 insert mutation were responsible for GDD (7). However, clinical treatment is restricted to surgical operations and symptomatic therapy.

Preliminary studies identify a pivotal effect of ANO5 on bone homeostasis. ANO5 promoted osteoclast differentiation through Nfatc1/AKT signaling pathway (8). While ANO5 knockout mice targeted exon 1–2 or exon 11–12 suppresses osteoclast activity (9, 10). Consistent with elevated serum alkaline phosphatase (ALP) level in GDD patients, *ANO5* knockdown resulted in abnormal enhancement of osteogenic activity (11). Previously, we reported p.Cys360Tyr mutation in a Chinese GDD family for the first time and generated an *Ano5* knock-in mouse model carrying p.Cys360Tyr mutation using Repeats (CRISPR)/CRISPR-associated protein 9 (Cas9) technology (11, 12). Homozygous knock-in mice (*Ano5^KI/KI^*) exhibited increased bone mineral density (BMD) and bone mineral content (BMC) in both mandibles and diaphysis of the limb bones, as well as increased bone fragility. Additionally, we found *Ano5* mutation led to increased osteogenesis decreased osteoclastogenesis (12). However, the specific mechanism of abnormal bone metabolism in GDD remains unclear.

Energy metabolism is indispensable for bone-related cells to maintain biological function. Production of adenosine triphosphate (ATP), the key factor for energy storage and transmission, is closely related to various biological processes, such as cell proliferation, apoptosis, and differentiation. Glucose, taken up by glucose transporter (GLUT), is the primary energy source and carbon in osteoblast (13). Despite sufficient oxygen, aerobic glycolysis is the main process of converting glucose to lactate to produce approximately 80% ATP in mature osteoblasts, closely related to concurrent osteogenesis and mineralization (14). Additionally, mitochondrial respiration is another key approach of energy metabolism, which plays a vital role in maintaining oxidative stress and ion homeostasis by exerting metabolic precursors and reactive oxygen species (ROS). PTEN-induced kinase 1 was a requisite during osteoblast differentiation through promoting a healthy pool of functional mitochondria (15). The oxidative phosphorylation (OXPHOS) system containing five enzyme complexes is the core of mitochondrial respiratory to generate ATP through the electron transport chain (ETC) (16). Robust mitochondrial biogenesis and supercomplex formation were observed during osteoblast differentiation, accompanied by increased ATP production and decreased mitochondrial stress (17).

It’s worth noting that mitochondria are extremely abundant in mature osteoclast, which guarantees sufficient energy provision to support osteoclast fusion and bone absorption (18). RANKL stimulated mitochondrial respiration during osteoclast differentiation through transcriptional regulation of PPARGC1B and estrogen receptor–related receptor α (ERRα) (19). Deletion components of OXPHOS complex or mitochondrial transcription factor TFAM induced osteoclast apoptosis and suppressed osteoclast maturation. Metabolic profiles for osteoblast and osteoclast are closely associated with the balance between bone formation and resorption.

AMP-activated protein kinase (AMPK) is a classical cellular energy sensor to regulate ATP production activated by falling energy status (20). AMPK signaling dysregulation is linked with systematic diseases, such as cancer, diabetes, muscle function, and aging health. AMPK directly phosphorylates mitochondrial fission factor (MFF) to regulate mitochondrial fission through dynamin-like protein DRP1 (21). Lack of AMPK α/β1/β2 subunits lead to defects in mitochondrial biogenesis and function in muscle via attenuating peroxisome proliferator-activated receptor-γ coactivator-1-α (PGC-1α) expression (22). AMPK also enhances glycolysis flux through activating PFK1, a rate-limiting enzyme in glycolysis, which is required for alveolar regeneration (23). Mice lacking of AMPKα exhibits reduced bone mass by retrained osteogenesis (24). While AMPK inhibits osteoclast differentiation and mitigates bone loss (25). A potential role for the metabolic regulation of AMPK in GDD has yet to be explored.

Herein, we investigate the effect and mechanism of glucose metabolism regulated by AMPK on abnormal bone remodeling of GDD. Elevated glycolysis and mitochondrial respiration were responsible for enhanced osteogenesis in *Ano5^KI/KI^* osteoblast. Whereas *Ano5* mutation inhibited mitochondrial function in osteoclast. Furthermore, we found *Ano5* mutation upregulated phosphorylation of AMPK, in turn, disturbing AMPK could rescue GDD bone abnormal phenotype by abrogating bone formation and stimulating osteoclastogenesis. Our study revealed that the chief mechanism of GDD was attributed to energy metabolism disorder mediated by AMPK, which rationalized therapeutic targeting of AMPK inhibitor for clinical management of GDD.

## 2 Materials and methods

### 2.1 Mouse model of *Ano5^Cys360Tyr^* knock-in and drug administration

*Ano5^Cys360Tyr^* knock-in mouse model (c.1080G>A) was generated by introducing tyrosine at codon 360 through Clustered Regularly Interspaced Palindromic Repeats (CRISPR)/CRISPR-associated protein 9 (Cas9) technology. C57BL/6 female mice and KM mouse strains were used as embryo donors and pseudopregnant adoptive mothers respectively. Specifics about the construction strategies of the Ano5 knock-in mouse model were previously described. DNA was extracted from mouse tail and then polymerase chain reaction (PCR) was performed (forward primer: 5’-GCTTAGGTCTTCTACATCGGGCTGT-3’ and reverse primer: 5’-ATCCCCATGAAGAGCGCAAAGAACA-3’). Then Sanger sequencing was used to clarify genotype. Mice were raised in a specific pathogen-free room at a temperature of 25°C with a 12-hour light-dark cycle and were provided with standard feed and libitum. For examining the effect of AMPK inhibitor on bone phenotype of GDD, 8-week-old male *Ano5^KI/KI^* mice were given AMPK inhibitor CC (5mg/kg, MCE, HY-13418) by gavage three times a week for four weeks, control group was given NaCl. All animal experimental protocols were approved by the Animal Ethics Committee of Capital Medical University Affiliated Beijing Stomatological Hospital (Ethics Review Number: KQYY-202012-005).

### 2.2 Cell cultures

Mouse calvarial osteoblast (mCOB) was isolated from the calvarial of postnatal day 3–5 mice through digesting with trypsin-EDTA (0.25%, Thermo, 25200056) and collagenase (3mg/mL, Sigma, C0130) at a mixture of 1:1 and cultured as previously described. Osteogenic medium (αMEM; Gibco 12561056) containing 10% FBS (Thermo, 10099141C), 4 μg/ml dexamethasone (Sigma, D4902), 50 mg/ml ascorbic acid (Sigma, A5960) and 4 mM β-glycerophosphate (Sigma-Aldrich, G9422) were used to induce osteoblast differentiation. Bone marrow macrophage (BMM) was isolated from tibias and femurs of 8-week-old male mice and cultured in αMEM with 10% FBS for 24 hours. Subsequently, Non-adherent cells were collected and suspended in αMEM containing 10% FBS and 30ng/mL macrophage-colony stimulation factor (M-CSF, Novoprotein, CK02) to stimulate BMM proliferation. After 3 days, 100ng/mL receptor activator of NF-κB ligand (RANKL, Novoprotein, CJ94) was used to induce osteoclast differentiation and maturation.

### 2.3 ALP analysis and AR staining

ALP activity was measured with RIPA lysis of the mCOB at 7 days of osteogenic differentiation and OD values were quantified at 520 nm according to the instruction of alkaline phosphatase assay kit (Nanjing Jiancheng Bioengineering institute, A059-2). Simultaneously, ALP staining was performed by using BCIP/NIBT alkaline phosphatase color development kit (Beyotime, C3206). Alizarin red staining solution (1mg/mL, Sigma-Aldrich) was added to the 75% ethanol-fixed osteoblasts at 21 days of the induction. Mineralized nodules were incubated with cetylpyridinium chloride for 30 minutes and absorbance was determined at 562 nm to detect calcium content.

### 2.4 TRAP and Phalloidin staining

BMMs at a density of 1×10^4^ cells/well were plated into 24-well plate. After 7-day induction, osteoclasts were fixed with 4% paraformaldehyde and then TARP staining (Sigma Aldrich, 387A) was used to detect the number of TRAP-positive osteoclasts (more than 3 nuclei). F-actin ring was stained by using cytopainter phalloidin-iFlour 488 (Abcam, ab176753) for 40 minutes and cell nucleus was stained under 4,6-diamino-2-phenylindole (DAPI) for 5 minutes. After washing with PBS, the cells were observed under a fluorescence microscope (Olympus BX51, Tokyo, Japan).

### 2.5 Measurement of extracellular acidification rate and oxygen consumption rate

Seahorse XF mitochondrial stress test was performed to assess mitochondrial function in mCOB and BMMs by measuring oxygen consumption rate. mCOBs at 3×10^4^ cells/well or BMMs at 5×10^4^ cells/well were seeded in poly-Llysine-coated XF24 V7 microplate (Seahorse Bioscience). AMPK inhibitor CC treated for 24 hours. On the day of measurement, cells were cultured in XF medium with glucose (1.0 mol), 0.5 mL Pyruvate solution (100 mM), and glutamine (200 mM) for 1 hour in a CO_2_-free incubator at 37°C before loading. Oligomycin (1.5 μM final), FCCP (3mM), antimycin A (0.5 μM), and rotenone (0.5 μM) were used to measure OCR (pmol/min). Data were normalized to the cell numbers or the protein content in each well.

### 2.6 Measurement of ATP

Intracellular ATP level was detected by the ATP Assay Kit according to instructions. mCOBs and BMMs seeded in 6 well plate at 5×10^5^ cells/well density were lysed and supernatants were collected by centrifugation at 12000*g* for 5 min. RLU value was measured with the chemiluminescence (luminometer) model of microplate reader after mixing detection working solution and supernatant. ATP level was calculated according to standard curve and normalized to protein content.

### 2.7 Lactate content and Lactate dehydrogenase activity measurements

mCOBs were lysed by PBS with 0.1% Triton-X 100 and supernatant were collected to measure intracellular lactate levels through using Lactic Acid assay kit to determine OD value at 450 nm. Meanwhile, CheKine™ Lactate dehydrogenase Activity Assay Kit (Micromethod) was used to detect LDH activity of mCOBs.

### 2.8 Mitochondrial membrane potential measurement

BMMs seeded in 6 well plate at 5×10^5^ cells/well density were induced with M-CSF and RANKL for 5 days. Cells were collected after trypsin digestion and mitochondrial membrane potential was detected with JC-1 assay kit (Beyotime, C2006) according to instructions. JC-1 was aggregated in the mitochondrial matrix to form JC-1 polymer with high mitochondrial membrane potential. While JC-1 exists as a monomer at low mitochondrial membrane potential. Flow cytometry was used to detect JC-1 monomer with excitation light at 490nm and emission light at 530nm, and JC-1 polymer with excitation light at 525nm and emission light set at 590nm.

### 2.9 qRT-PCR

The total RNA of mCOBs or BMMs was extracted using Trizol reagent (Invitrogen, 15596026) and 500 ng of RNA was reverse transcribed with PrimeScript™ RT reagent Kit (Takara, RR037A). qRT-PCR reactions containing 1 μl cDNA, 5 μM primer (Sangon Biotech), 10 μl MagicSYBR Mixture (CWbio, CW3008), and 7 μl RNase-free water were performed with Bio-Rad thermocycler (BioRad) following corresponding thermal cycling program. Data were analyzed using the 2^−ΔΔCt^ method normalizing to the internal control of Actb. PCR primer sequences are listed in **Supplementary Table 1**.

### 2.10 Western blot

Proteins of mCOBs or BMMs were extracted using PIPA buffer (Applygen, C1053) added with protease inhibitor (Beyotime, P1006). Coomassie brilliant blue was used to quantify protein concentration according to the OD value at 595 nm. 20 μg Protein was separated on 4-20% (w/v) sodium dodecyl sulfate polyacrylamide gels and transferred onto transferred to a PVDF membrane. After blocking with 5% nonfat milk for 1 hour, the membrane was incubated with primary antibodies overnight at 4°C. The membranes were then washed 3 times with TBST and incubated with HRP-conjugated anti-rabbit or anti-mouse IgG secondary antibody for 1 hour at room temperature. ChemiDoc Touch Imaging System (Bio-Rad) was used to capture blot images and quantify band’s density. The housekeeping protein was detected with ACTB or Tubulin. All primary antibodies used are listed in **Supplementary Table 2**.

### 2.11 Immunofluorescence

After fixation with 4% paraformaldehyde for 25 minutes, mCOBs were permeabilized and blocked with QuickBlock™ immunostaining blocking solution (Beyotime, P0260) for 15 minutes. The cells were incubated with primary antibodies overnight at 4°C, washing with PBS, incubated with 594-conjugated Goat anti-Rabbit secondary antibody for 1 hour. Cytoskeleton were labeled with Phalloidin staining and cell nuclei were stained with DAPI. ZEISS ZEN2 was used to quantify Immunofluorescence density.

### 2.12 Cell proliferation assay

The proliferative ability of mCOBs was tested by cell counting kit-8 (CCK-8) assay (Dojindo, Tokyo, Japan) according to the manufacturer’s protocol. Cells were seeded into 96-well plates at a density of 5 × 10³ cells/well. At days 1, 2, 3, and 4, the complete medium was changed to serum-free medium containing 10 μl CCK-8 reagent and incubated with the cells at 37°C for 2 hours. Absorbance was measured at 450 nm using a microplate reader. Meanwhile, the viability of mCOBs after 7 and 14 days of osteoblast differentiation was also measured. Cell viability was calculated as follows: cell viability = (OD_treated_ - OD_blank_)/(OD_untreated_ - OD_blank_).

### 2.13 μCT

μCT was used to observe the morphology and structure of mandible, tibia, and femur of mice at a resolution of 7 μm. The region from 50–100 nm below the growth plate was selected for 3-dimensional histomorphometric analysis to determine trabecular bone morphometric parameters, including bone volume/tissue volume (BV/TV), trabecular number (Tb.N), trabecular separation/spacing (Tb.Sp), trabecular thickness (Tb.Th), and bone surface/bone volume (BS/BV). Middle tibia or femur was chosen to measure cortical bone thickness (Ct.Th), bone mineral density (BMD), bone mineral content (BMC), and BS/BV. For mandibular scanning, the region of interest was selected around the first molar and the mandibular image was captured on the vertical section of the center fossa of the mandibular first molar.

### 2.14 ELISA

Serum was collected through retro-orbital bleeding from mice. Procollagen I N-terminal propeptide (PINP) and type I collagen cross-linked C-telopeptide (CTX) in the serum were measured by using mouse PINP ELISA Kit (Elabscience, E-EL-M0233) and mouse β-CTx ELISA Kit (Elabscience, E-EL-M0372) following the manufacturer’s protocol. Mini sample mouse receptor activator of nuclear factor kappa B ligand (RANKL) ELISA Kit (Elabscience, E-MSEL-M0084) and mouse osteoprotegerin (OPG) ELISA Kit (Elabscience, E-EL-M0081) was used to detect serum RANKL and OPG level.

### 2.15 Bone biomechanical properties

Freshly harvested mouse tibiae were dissected, cleaned of soft tissue, and stored in NaCl to maintain hydration. The bones were positioned horizontally on a three-point bending fixture with the anterior surface facing upward. The span length between the lower supports was adjusted to 12 mm to ensure mid-diaphysis loading. A universal mechanical testing machine was used to apply a vertical displacement-controlled load at the midpoint of the tibia at a rate of 1 mm/min until failure. The displacement-load curve, the elastic modulus, maximum load, and fracture load were recorded. After measurement of diameter of the tibial fracture, the maximum stress and fracture stress were calculated according to the formula S=F×L/[D^3×3.1416/32] (S: stress, F: load, D: diameter).

### 2.16 Transmission electron microscope detection

The Ward’s triangle of femurs of 12-week-old wildtype and *Ano5^KI/KI^* male mice was fixed with 2.5% glutaraldehyde and decalcified for 4 weeks. According to TEM preparation instruction, the samples were fixed again with 1% osmic acid, dehydrated, and embedded. 70–90 nm sections made with LEICA EM UC7 ultramicrotome were stained with lead citrate uranyl acetate and observed under HT7700 Exalens TEM.

### 2.17 Histomorphometry

Tibia and femur of mice were collected. After fixation with paraformaldehyde for 24 hours, 10% EDTA was used for decalcification until there was no resistance when the needle was inserted. Then, conventional dehydration, paraffin embedding, and longitudinal section at a thickness of 5 μm were carried out according to the established procedures (12). H&E staining was performed to observe morphology and structure. TRAP staining was used to assess osteoclast formation according to the manufacturer’s instructions (Sigma-Aldrich, 387A-1KT). For immunohistochemistry experiment, paraffin section paraffin sections were incubated with primary antibodies, including anti-OCN (Affinity, DF12303) at a dilution of 1:100 and anti-NFATC1 (Santa cruz, sc-7294), at a dilution of 1:50. Images were acquired using an Olympus BX61 microscope (Olympus, Tokyo, Japan), and immunopositive areas were analyzed using Image-Pro Plus 6.0.

### 2.18 Glucose uptake detection

Glucose uptake was measured with Glucose uptake probe-Red (Dojindo, UP03) of the mCOB at days 0, 7, and 14 of osteogenic differentiation according to the manufacturer’s protocol. In brief, mCOB was pretreated in medium without glucose for 15 minutes. Then, glucose uptake probe (Ex/Em=545 nm/605 nm) was incubated for 15 minutes and Honchest was used to label nucleus. The fluorescence images were observed under a fluorescence microscope (Olympus BX51, Tokyo, Japan) and immunofluorescence density was measured.

### 2.19 Statistics and reproducibility

Student’s 2-tailed t tests were used when determining the statistical differences between the 2 groups; Comparisons between two groups were carried out using an unpaired, two-tailed Student’s t test using GraphPad Prism 8.0.2 software (GraphPad Software, La Jolla, CA, USA). For the comparison in more than two groups, one-way analysis of variance (ANOVA) was used. While comparing multiple groups, we used one-way ANOVA followed by the Dunnett’s test for multiple comparisons. To analyze the effect of two variables and the interaction of those variables, we used two-way ANOVA followed by the Dunnett’s test for multiple comparisons. All data are presented as the mean ± standard error of the mean (SEM). Quantitative data were obtained from at least three (*n*≥ 3) independent experiments to ensure reproducibility.

## 3 Results

### 3.1 *Ano5^Cys360Tyr^* mutation promoted glucose metabolism in osteoblast

**Figure 1:**
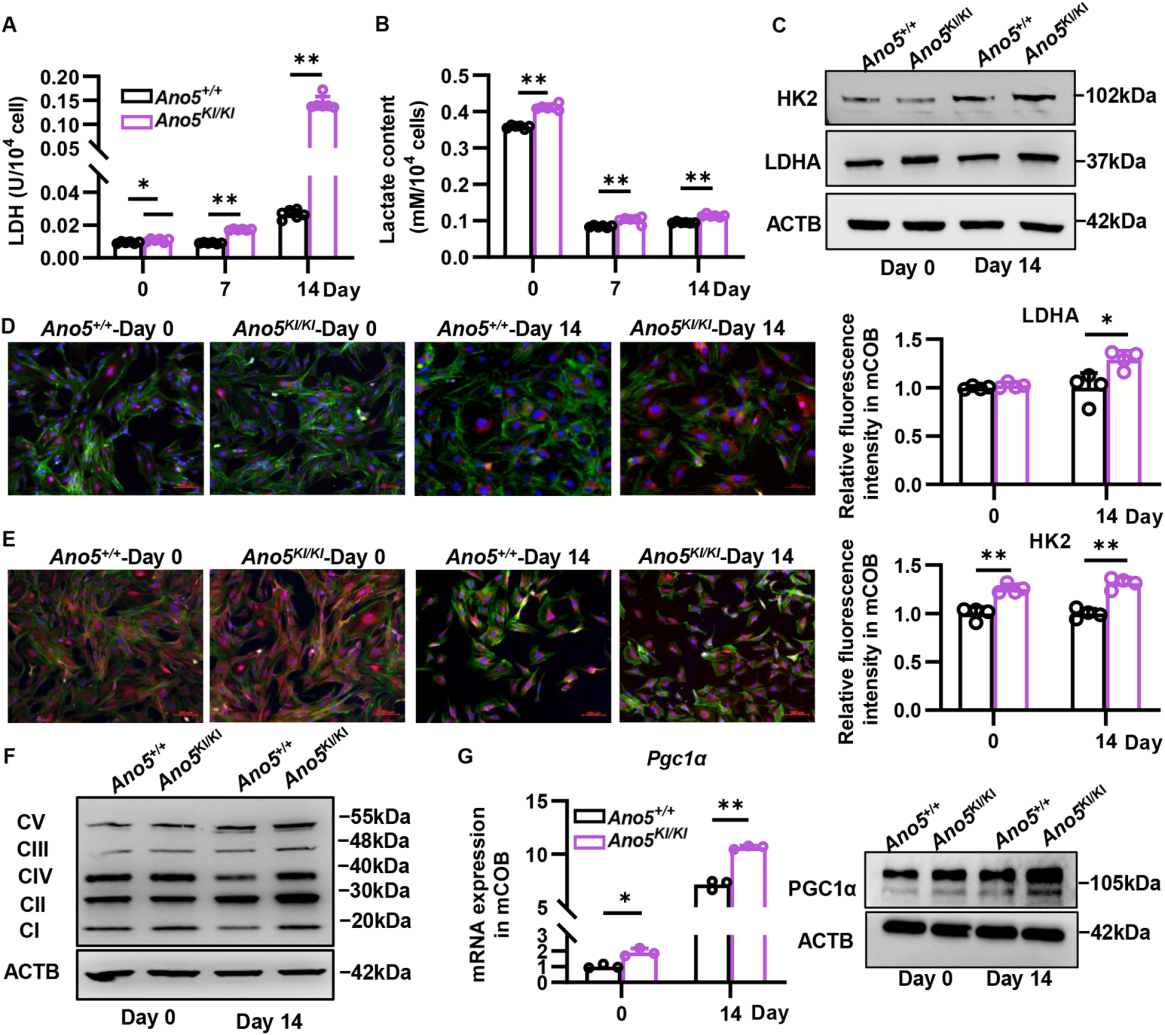
*Ano5^Cys360Tyr^* mutation upregulates glucose metabolism in osteoblasts. **(A, B)** LDH activity **(A)** and lactate content **(B)** in *Ano5^+/+^* and *Ano5^KI/KI^* mCOB at different osteogenic induction; **(C)** Relative expression of HK2 and LDHA by Western blot in *Ano5^+/+^* and *Ano5^KI/KI^* mCOB at days 0 and 14; **(D, E)** Immunofluorescence images (red) and relative quantitative analysis of HK2 **(D)** and LDHA **(E)** in *Ano5^+/+^* and *Ano5^KI/KI^* mCOB, Phalloidin staining (green) representing cytoskeleton, and DAPI indicating cell nucleus, bar=200 μm; **(F)** Immunoblotting analysis of OXPHOS complex (CI-NDUFB8, CII-SDHB, CIII-UQCRC2, CIV-MTCO1, CV-ATP5A) and ACTB in mCOB; **(G)** qRT-PCR (left) and immunoblotting (right) analysis of PGC1α in *Ano5^+/+^* and *Ano5^KI/KI^* mCOB at days 0 and 14. Data are presented as mean ± SEM. Statistic significances are determined by t-tests, with *P < 0.05, **P < 0.01.

Increased glycolysis is a common and essential feature of osteoblast differentiation, which is highly dependent on metabolic enzymes. Lactate dehydrogenase A (LDHA), one of the rate-limiting enzymes in glycolytic process, is responsible for converting pyruvate to lactate. We found that LDHA activity of *Ano5^KI/KI^* mCOB was usually increased with osteoblast differentiation and was more than five times than that of the wild group, especially at day 14 (**Figure 1A**). Correspondingly, the lactate content in intracellular was higher in intracellular of *Ano5^KI/KI^* mCOB than *Ano5^+/+^* group, while without a significant difference in supernatant (**Figure 1B**; **Supplementary Figure S1A**). Although LDHA expression was comparable between *Ano5^KI/KI^* and *Ano5^+/+^* mCOB at day 0, *Ano5^Cys360Tyr^* mutation obviously promoted LDHA expression in mature osteoblast (**Figures 1C, D**). Furthermore, Western blot and immunofluorescence exhibited that hexokinase2 (HK2) was significantly upregulated (**Figures 1C, E**; **Supplementary Figure S1B**), which indicated *Ano5* mutation stimulated glycolysis in osteoblast.

**Figure 2:**
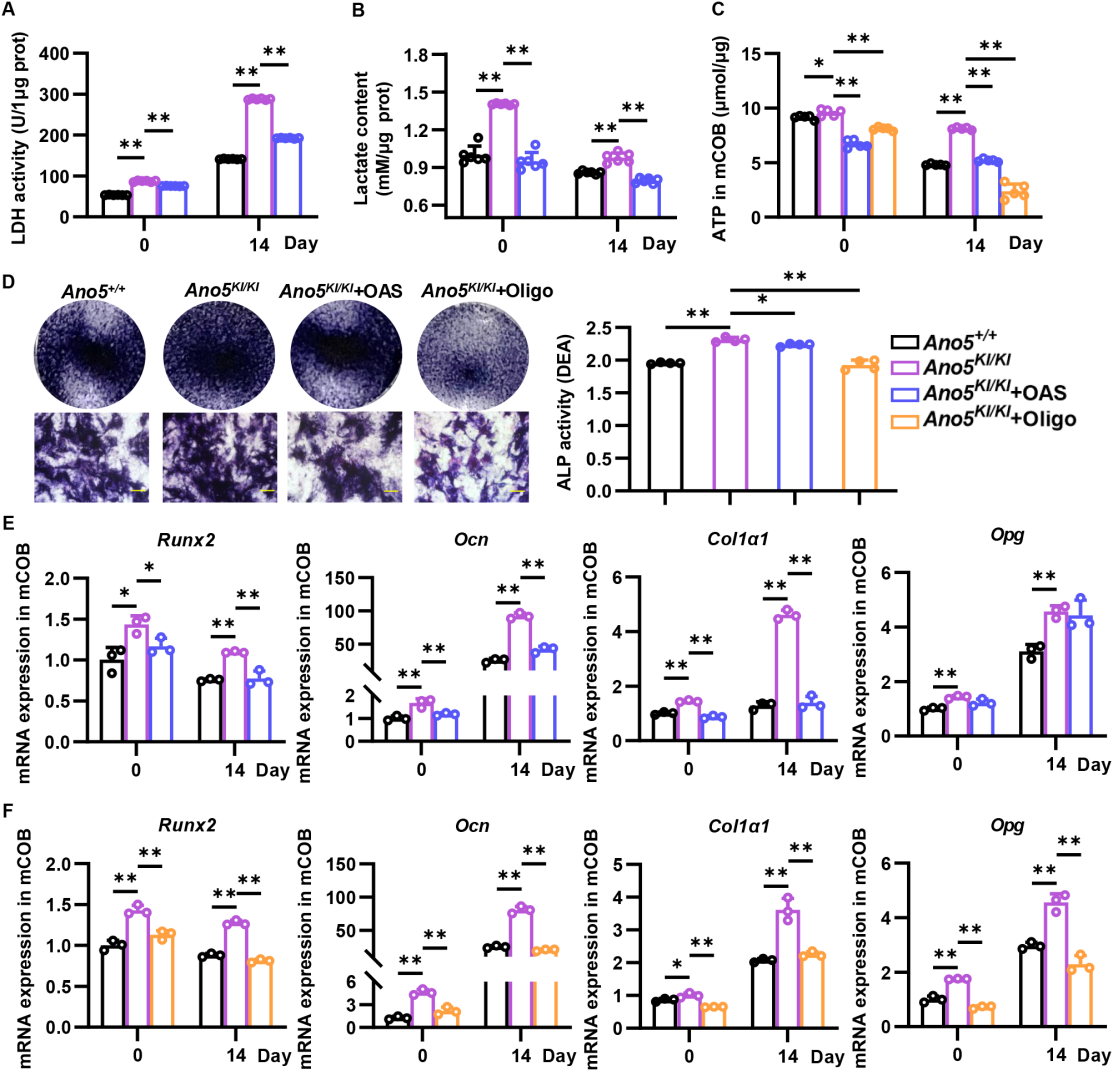
Inhibiting glycolysis and mitochondrial respiration attenuated osteogenesis of *Ano5^KI/KI^* mCOB. **(A, B)***Ano5^KI/KI^* mCOB was treated with OAS LDH activity **(A)** and lactate content in *Ano5^+/+^*, *Ano5^KI/KI^*, and *Ano5^KI/KI^* mCOB treated with OAS at days 0 and 14; **(C)** Intracellular ATP content in *Ano5^+/+^*, *Ano5^KI/KI^*, *Ano5^KI/KI^* mCOB treated with OAS or Oligo at days 0 and 14; **(D)** ALP staining (bar=100 μm) and activity analysis of *Ano5^+/+^*, *Ano5^KI/KI^*, *Ano5^KI/KI^* mCOB treated with OAS or Oligo at day 7; **(E, F)** qRT-PCR analysis of *Runx2*, *Ocn*, *Col1α1*, and *Opg* in *Ano5^+/+^*, *Ano5^KI/KI^*, *Ano5^KI/KI^* mCOB treated with OAS **(E)** or Oligo **(F)**. Data are presented as mean ± SEM. Statistic significances are determined by one-way ANOVAs with Dunnett’s multiple comparison tests, with *P < 0.05, **P < 0.01.

Additionally, mitochondrial respiration is important for providing cellular energy and regulating osteoblast activity. Mito-tracker staining showed the number of mitochondria was slightly increased in *Ano5^KI/KI^* mCOB compared with wildtype group (**Supplementary Figure S1C**). Electron transporting respiratory chain is the core to maintain mitochondria function. Western blot exhibited *Ano5* mutation upregulated the expression of OXPHOS complex, especially after 14-day induction (**Figure 1F**; **Supplementary Figure S1D**). The respiratory chain is a major source of intracellular ROS, which declined in mature *Ano5^KI/KI^* mCOB (**Supplementary Figure S1E**). Furthermore, PGC1α that is the master regulator of mitochondrial biogenesis and function in osteoblast was significantly increased in *Ano5^KI/KI^* mCOB (**Figure 1G**; **Supplementary Figure S1D**). Glycolysis and mitochondrial respiration jointly generate sufficient ATP in osteoblast, and we observed a about 50% increase in intracellular ATP content caused by *Ano5* mutation at late osteogenic stage (**Figure 2C**).

### 3.2 Inhibiting energy metabolism rescued osteogenesis of *Ano5^KI/KI^* mCOB

To reveal the effect of energy metabolism dysregulation on enhanced osteogenesis of GDD, *Ano5^KI/KI^* mCOB was exposed to oxamic acid sodium (OAS), a specific inhibitor of LDHA enzyme. 10mM OAS, which was selected according to CCK8 analysis, could significantly suppress LDHA activity and lactate content in *Ano5^KI/KI^* mCOB both at day 0 and day 14 after osteogenic induction (**Figures 2A, B**; **Supplementary Figure S2A**). Additionally, Oligomycin (Oligo), an inhibitor of H^+^-ATP synthetase, was utilized to break oxidative phosphorylation and electron transport chains. We found not only OAS but also Oligo restrained ATP production of *Ano5^KI/KI^* mCOB (**Figure 2C**). Fundamentally, the enhanced ALP activity of *Ano5^KI/KI^* mCOB, a marker of early osteoblast differentiation, was attenuated by OAS, accompanied by reduction of *Ocn*, *Col1α1*, *Runx2*, and *Opg* level (**Figures 2D, E**). Exposure to Oligo also restrained osteogenesis caused by *Ano5^Cys360Tyr^* mutation (**Figures 2D, F**). The above results indicated that energy metabolism dysregulation caused by *Ano5* mutation plays a vital role in enhancing bone formation of GDD.

### 3.3 *Ano5^Cys360Tyr^* mutation induced glucose metabolism through activating AMPK in osteoblast

**Figure 3:**
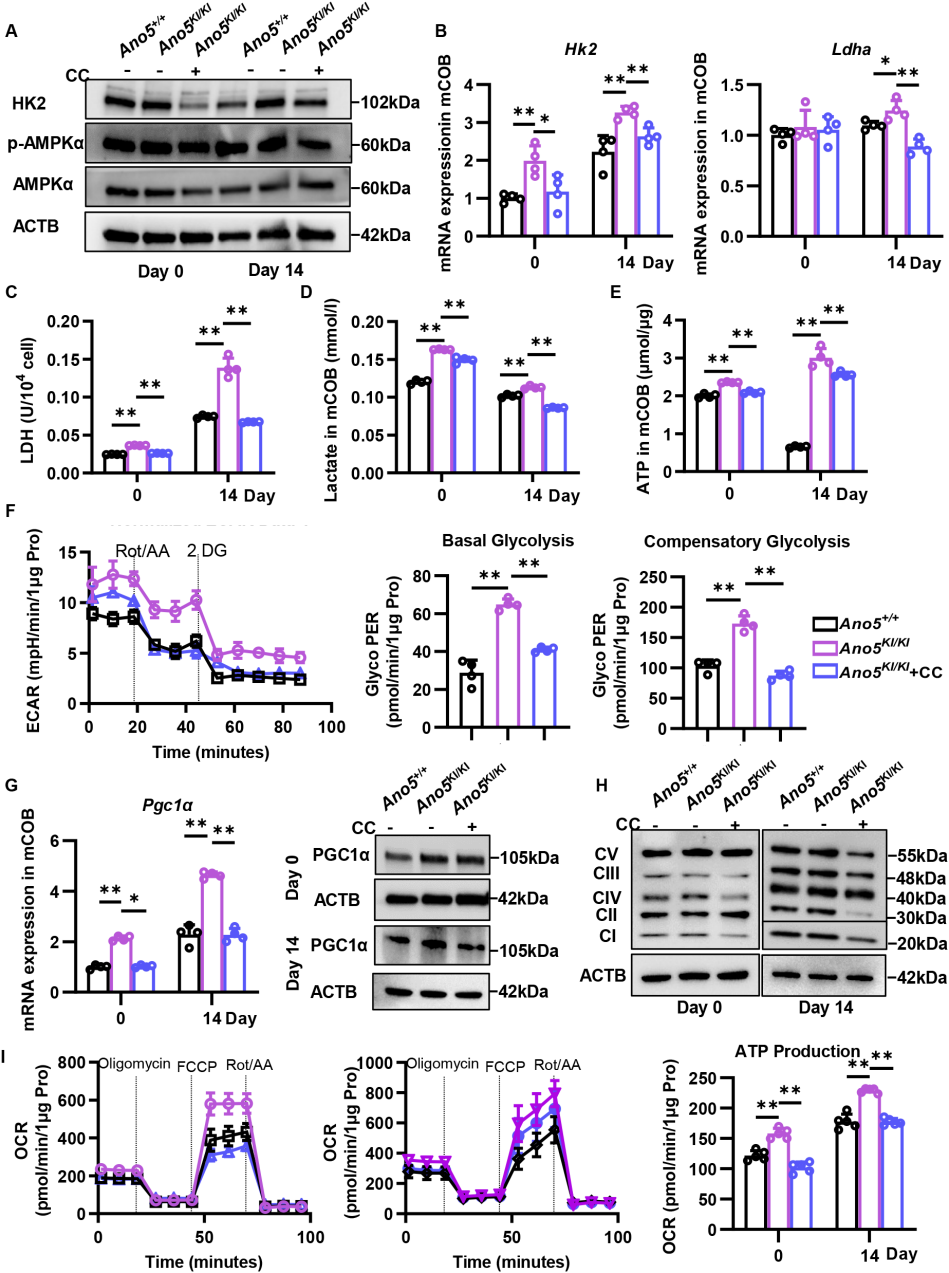
AMPK activation is responsible for enhanced glucose metabolism in *Ano5^Cys360Tyr^* osteoblast. To examine the effect of AMPK on abnormal glucose metabolism, *Ano5^KI/KI^* mCOB was treated with 5μM CC. **(A)** Immunoblotting analysis of HK2, p-AMPKα/AMPKα, and ACTB at days 0 and 14 of osteogenic induction; **(B)** qRT-PCR analysis of *Hk2* and *Ldha*; **(C–E)** LDH activity **(C)**, lactate content **(D)**, and ATP content **(E)** in mCOB; **(F)** Seahorse XF glycolysis rate examination at day 14, and relative quantitative analysis of basal and compensatory glycolysis ability according to proton efflux rate (PER); **(G)** qRT-PCR (left) and immunoblotting (right) analysis of PGC1α; **(H)** Immunoblotting analysis of OXPHOS complex and ACTB; **(I)** Seahorse XF mitochondrial stress examination at days 0 and 14, ATP production ability according to oxygen consumption rate (OCR). Data are presented as mean ± SEM. Statistic significances are determined by one-way ANOVAs with Dunnett’s multiple comparison tests, with *P < 0.05, **P < 0.01.

AMPK is a crucial energy sensor under various metabolic stresses to maintain metabolic homeostasis. AMPK contains catalytic α subunit and regulatory β and γ subunits and is activated by phosphorylation at Thr172 of α subunit. Western blot exhibited a higher ratio of phosphorylated AMPKα to total AMPK in *Ano5^KI/KI^* mCOB than *Ano5^+/+^* group (**Figure 3A**). More critically, AMPK inhibitor compound C down-regulated the expression of HK2 (**Figures 3A, B**; **Supplementary Figure S2B**). Although the mRNA expression of LDHA was only suppressed at day 14, intracellular LDHA activity companied with lactate content were also declined in *Ano5^KI/KI^* mCOB with Compound C stimulation (**Figures 3C, D**). Seahorse detection of glycolytic rate is a more powerful method to observe the energy metabolism process according to detecting extracellular acidification rate (ECAR). *Ano5* mutation elevated basal glycolysis and compensatory glycolysis in osteoblast, which were attenuated by Compound C stimulation (**Figure 3F**). AMPK not only takes part in glycolysis but also mitochondrial respiration through regulating PGC1α^6^. qRT-PCR and Western blot exhibited that Compound C decreased PGC1α expression in *Ano5^KI/KI^* mCOB, accompanied with reduced OXPHOS complex expression (**Figures 3G, H**; **Supplementary Figure S2B, D, E**). Importantly, Seahorse analysis showed that augmented mitochondrial respiration ability in *Ano5^KI/KI^* mCOB, including basal respiration and ATP production, was reversed by AMPK inhibitor both at day 0 and day 14 (**Figure 3I**; **Supplementary Figure S2C**). AMPK inhibitor also decreased ATP level in *Ano5^KI/KI^* mCOB (**Figure 3E**), which may be attributed to both glycolysis and mitochondrial respiration. Energy metabolism dysregulation of *Ano5^KI/KI^* osteoblast was mainly determined by excessive activation of AMPK signaling, while its role in enhanced osteogenesis remains to be further elucidated.

### 3.4 Ablation of AMPK signaling mitigated the enhanced osteogenesis in GDD

**Figure 4:**
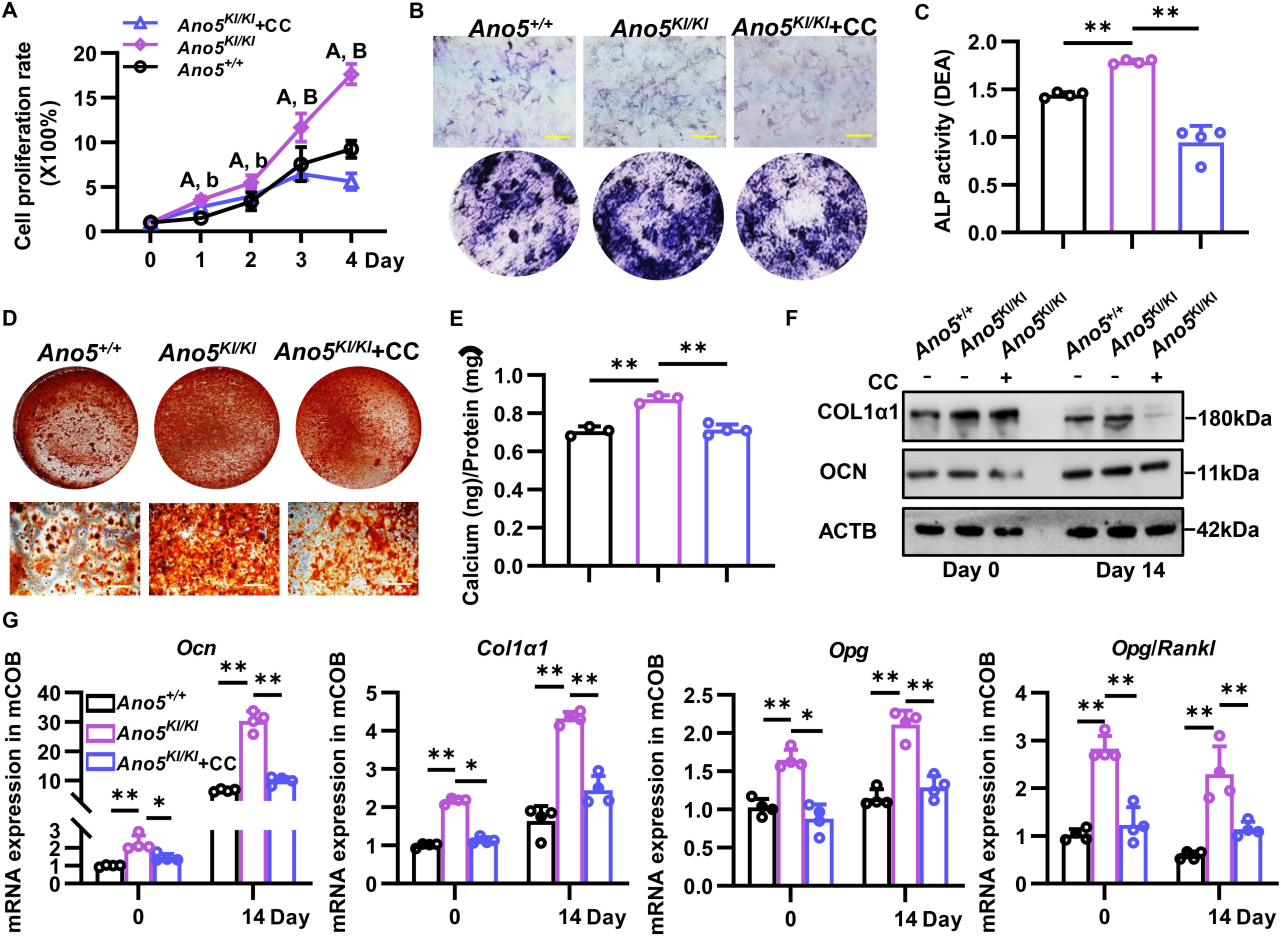
AMPK inhibitor attenuates osteogenesis of *Ano5^KI/KI^* mCOB. To examine the effect of AMPK on abnormal glucose metabolism, *Ano5^KI/KI^* mCOB was treated with 5μM CC. **(A)** Cell proliferation analysis by CCK8 detection of mCOB at days 0, 1, 2, 3, and 4 without osteoblast differentiation (*^A^P* < 0.01 representing *Ano5^KI/KI^ vs Ano5^+/+^*, *^b^P* < 0.05 and *^B^P* < 0.01 representing *Ano5^KI/KI^*+Compound C *vs Ano5^KI/KI^*); **(B, C)** ALP staining [**(B)**, bar=100 μm] and activity analysis **(C)** at day 7 of osteogenic induction; **(D, E)** Alizarin Red S staining [**(D)**, bar=100 μm] and histogram of the corresponding calcium-binding levels in the mineral nodules **(E)** at day 21; **(F)** Immunoblotting analysis of Col1α1, Ocn, and ACTB; **(G)** qRT-PCR analysis of *Ocn*, *Col1α1*, *Opg* and *Opg/Rankl*. Data are presented as mean ± SEM. Statistic significances are determined by one-way ANOVAs with Dunnett’s multiple comparison tests, with *P < 0.05, **P < 0.01.

Then, we examined whether *Ano5* mutation-inducible AMPK signaling contributed to elevated osteogenesis of GDD. Osteoblast proliferation, differentiation, and matrix mineralization are crucial for bone formation. It’s known that AMPK regulates proliferation of cancer cells, vascular smooth muscle cells, and lymphoma cells. Our previous study showed that ANO5 mutation significantly promoted osteoblasts proliferation. CCK8 analysis exhibited AMPK inhibitor obviously blocked the proliferation of *Ano5^KI/KI^* mCOB accompanied with declined *Ki67* (**Figure 4A**; **Supplementary Figures S2F, G**). Not only GDD patients but also *Ano5* deficiency mice are featured with elevated serum ALP level. Our study revealed that blocking AMPK signaling mitigated ALP activity of *Ano5^KI/KI^* osteoblast (**Figures 4B, C**). AR staining showed a declined formation of extracellular mineral nodules in *Ano5^KI/KI^* group with Compound C treatment accompanied with low calcium content (**Figures 4D, E**). Furthermore, AMPK inhibitor attenuated the expression of OCN that could combine with calcium to regulate matrix mineralization (**Figure 4F**; **Supplementary Figure S2H**). Upregulation of COL1α1, the main collagen type in bone, was also mitigated in *Ano5^KI/KI^* mCOB by AMPK inhibitor (**Figure 4F**; **Supplementary Figure S2H**). OPG and RANKL synthesized by osteoblast play a vital role in balance bone formation and resorption. Although the *Rankl* mRNA level showed no statistical differences between *Ano5^KI/KI^* mCOB and Compound C group, the expression of *Opg* and the ratio of *Opg* to *Rankl* were obviously rescued by AMPK inhibitor treatment (**Figure 4G**; **Supplementary Figure S2I**). Therefore, we speculated that increased glucose metabolism mediated by AMPK activation is responsible for enhanced bone formation in GDD.

### 3.5 *Ano5^Cys360Tyr^* mutation disturbed mitochondrial structure and function of osteoclast

**Figure 5:**
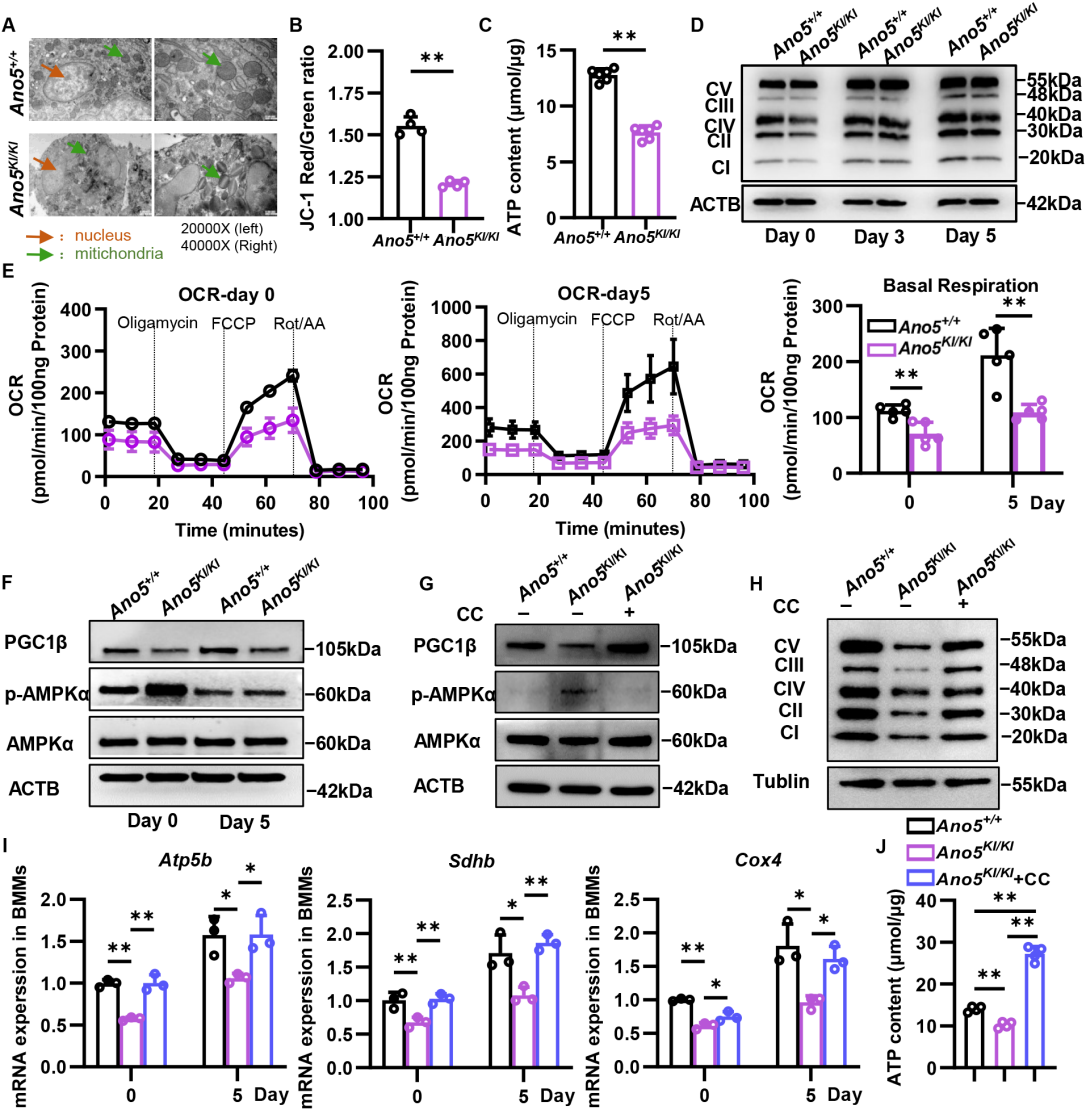
*Ano5^Cys360Tyr^* mutation interferes with AMPK-dependent mitochondrial function of osteoclast. **(A)** TEM scan of osteoclast in tibia of 12-week-old male mice, orange arrow indicating nucleus and green arrow indicating mitochondria; **(B)** Flow cytometry detecting JC-1 monomer (green) and polymer (red) of *Ano5^+/+^* and *Ano5^KI/KI^* osteoclast at day 5 of osteoclast differentiation; **(C)** Intracellular ATP level of *Ano5^+/+^* and *Ano5^KI/KI^* osteoclast at day 5 of M-CSF (30 ng/mL) and RANKL (100 ng/mL) induction; **(D)** Immunoblotting analysis of OXPHOS complex and ATCB at days0, 3, and 5; **(E)** Seahorse XF mito stress analysis at day 0 and 5, and histogram of basal respiration ability; **(F)** Immunoblotting analysis of PGC1β, p-AMPKα/AMPKα, and ACTB; **(G, H)** In order to examine the relation between AMPK activation and mitochondrial dysregulation of osteoclast, 5 μM AMPK inhibitor Compound C was administered to *Ano5^KI/KI^* BMM. **(G, H)** Immunoblotting analysis of PGC1β, p-AMPKα/AMPKα **(G)**, OXPHOS **(H)**, and ACTB at day 5; **(I)** Intracellular ATP content analysis at day 5; **(J)** qRT-PCR analysis of *Atp5b*, *Sdhb*, and *Cox4*. Data are presented as mean ± SEM. Statistic significances are determined by t-tests **(B, C)** and one-way ANOVAs with Dunnett’s multiple comparison tests **(E, I, J)**, with *P < 0.05, **P < 0.01.

Osteoclast differentiation induced by RANKL is accompanied by an increase in the size and number of mitochondria to produce sufficient ATP for osteoclast fusion and maturation. Mito-Tracker green fluorescence staining showed that the number of mitochondria in *Ano5^KI/KI^* osteoclasts was comparable to that of wildtype group at different stages of osteoclast differentiation (**Supplementary Figure S3A**). Transmission electron microscopy (TEM) scanning showed cell membrane of osteoclasts was intact in both *Ano5^+/+^* and *Ano5^KI/KI^* mice. However, the disappearance of bilayer membrane and abnormal crystallization in mitochondria matrix were observed in *Ano5^KI/KI^* osteoclast (**Figure 5A**). MMP is maintained by the asymmetric distribution of protons and ions on both sides of the mitochondrial inner membrane during the process of energy production. JC-1 staining in mature osteoclast exhibited that *Ano5* mutation resulted in an obvious reduction of MMP in mature osteoclast (**Figure 5B**). What’s more, MMP is indispensable for mitochondria to produce ATP. We also observed that *Ano5^Cys360Tyr^* mutation led to a significant decrease in intracellular ATP level (**Figure 5C**). The expression of OXPHOS complex was declined in both *Ano5^KI/KI^* BMMs and osteoclasts (**Figure 5D**). Subsequently, Mito-stress seahorse analysis revealed an almost 50% reduction in basal respiration, ATP production, and maximal respiratory capacity in *Ano5^KI/KI^* osteoclast compared with wildtype group (**Figure 5E**; **Supplementary Figure S3B**). These results suggested that *Ano5^Cys360Tyr^* mutation led to abnormal mitochondrial structure and respiratory function in osteoclast.

### 3.6 mitochondrial respiration dysregulation mediated by AMPK/PGC1β was responsible for decreased osteoclastogenesis in GDD

Another PGC1 family member PGC1β is specifically expressed in osteoclasts to regulate mitochondrial biosynthesis and function. PGC1β plays a positive role in osteoclast differentiation and bone resorption. To further explore the mechanism underlying declined mitochondrial respiration in *Ano5^KI/KI^* osteoclast, we investigated the effect of *Ano5^Cys360Tyr^* mutation on PGC1β expression. Western blot showed that PGC1β protein level was distinctly lower in *Ano5^KI/KI^* osteoclasts than *Ano5^+/+^* group (**Figure 5F**; **Supplementary Figure S3C**). Several studies showed that AMPK signaling is negatively correlated with osteoclast differentiation. Importantly, *Ano5^Cys360Tyr^* mutation stimulated phosphorylation of AMPKα in osteoclasts. There are few researches defining the effect of AMPK on energy metabolism of osteoclast. To corroborate the metabolic regulation by AMPK in osteoclast, Compound C was used to stimulate *Ano5^KI/KI^* osteoclasts. We found inhibiting AMPK could improve the protein level of PGC1β (**Figure 5G**; **Supplementary Figure S3D**). Compound C also increased OXPHOS complex expressions as confirmed by Western blot and qRT-PCR (**Figures 5H, I**; **Supplementary Figure S3E**). Meanwhile, ATP content mainly produced by mitochondrion in mature osteoclasts exhibited obvious elevation in *Ano5^KI/KI^* osteoclasts with Compound C stimulation elevation (**Figure 5J**).

**Figure 6:**
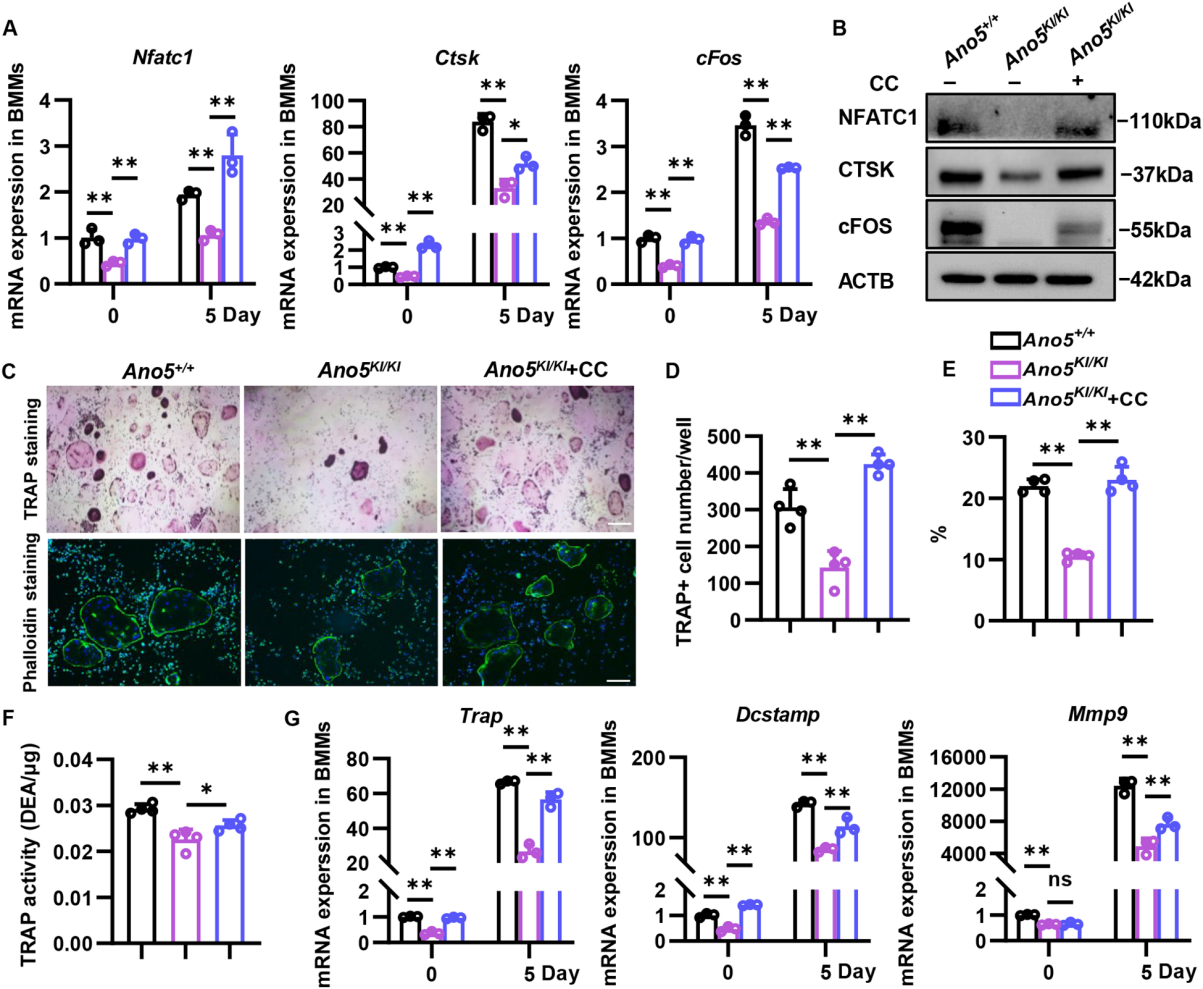
Blocking AMPK rescues osteoclastogenesis of *Ano5^KI/KI^* BMM. 5 μM AMPK inhibitor Compound C was administered to *Ano5^KI/KI^* BMM. **(A, B)** qRT-PCR **(A)** and immunoblotting **(B)** analysis of NFATC1, CTSK, and cFOS at days 0 and 5; **(C)** TRAP (above, bar=100 μm) and Phalloidin (bellow, green, bar=200 μm) staining at days 7 with DAPI labeling cell nucleus; **(D)** the number of TRAP-positive cells per well; **(E)** the percentage of nuclei in actin ring-positive osteoclasts to the total number of nuclei; **(F)** TRAP activity analysis of osteoclast at day 5 of RANKL stimulation normalized to protein content; **(G)** qRT-PCR analysis of *Trap*, *Dcstamp*, and *Mmp9*. Data are presented as mean ± SEM. Statistic significances are determined by one-way ANOVAs with Dunnett’s multiple comparison tests, with *P < 0.05, **P < 0.01.

Given that AMPK activation is responsible for abnormal mitochondrial function in *Ano5^Cys360Tyr^* osteoclasts, we then validated the relation between AMPK signaling and osteoclast differentiation. Compound C obviously elevated the expression of NFATC1 that is a crucial transcription factor in regulating osteoclast-related genes expression (**Figures 6A, B**). Additionally, decreased mRNA and protein levels of Ctsk and cfos were rescued (**Figures 6A, B**; **Supplementary Figure S3F**). TRAP staining showed that the number of mature osteoclasts was noticeably higher with Compound C treatment than *Ano5^KI/KI^* group, accompanied with increased TRAP activity (**Figures 6C, D, F**). Disruption of AMPK signaling also improved *Trap* transcription (**Figure 6G**). Furthermore, F-actin ring is indispensable for attachment of osteoclasts to the bone surface and formation of a sealed resorption vacuole. Phalloidin staining displayed that blocking AMPK promoted the formation of F-actin ring in *Ano5^KI/KI^* osteoclasts (**Figures 6C, E**). The expression of *Atp6v0d2* and *Dcstamp* involved in F-actin ring formation were also enhanced with Compound C treatment (**Figure 6G**; **Supplementary Figure S3G**). Besides multi-nuclear osteoclast maturation, protons and lytic enzymes secretion are indispensable for bone resorption. Chloride channel 7 (CLCN7) acts as a Cl^-^/H^+^ exchange function to be physiologically important for the acidic environment in sealed resorption vacuole and Mmp9 is vital for the degradation of collagen matrices (27). Declined *Clcn7* and *Mmp9* caused by *Ano5* mutation were rescued by AMPK inhibition (**Figure 6G**; **Supplementary Figure S3G**). The above results supply strong evidence that energy metabolism dysregulation mediated by AMPK activation is responsible for inhibited osteoclast activity in GDD.

### 3.7 Blocking AMPK rescued abnormal bone phenotype of GDD

**Figure 7:**
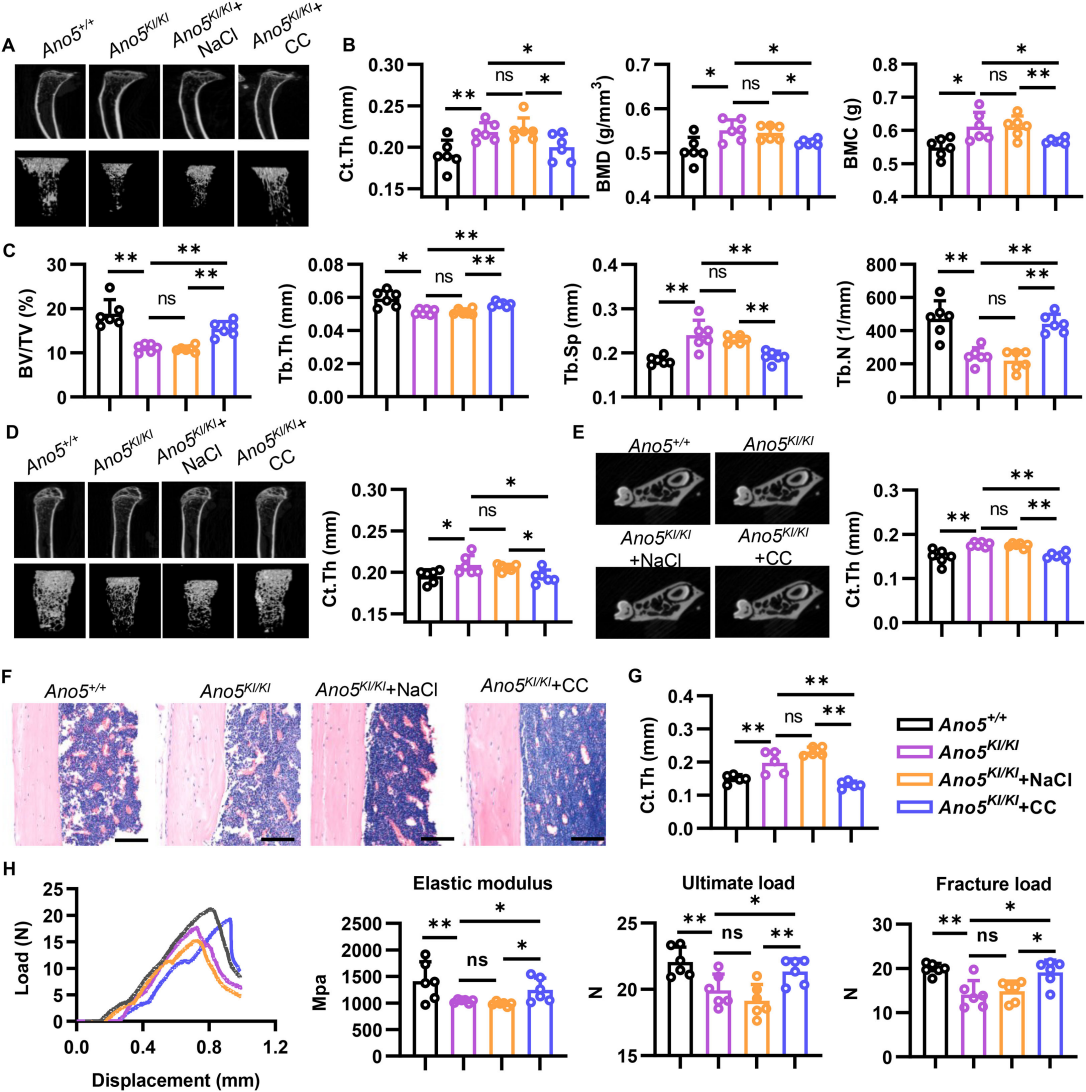
AMPK inhibitor effectively rescues bone metabolism of GDD. 12-week-old male mice were used to observe bone phenotype. **(A)** μCT images of vertical plane and trabeculae 3D reconstructions of tibia. **(B, C)** Quantification analysis of μCT analysis of cortical bone **(B)** and trabecula bone **(C)** of tibia; **(D)** μCT images of vertical plane and trabeculae 3D reconstructions of femur, and quantification analysis of cortical bone thickness; **(E)** μCT images of vertical plane of mandible and quantification analysis of palatal cortical bone thickness; **(F, G)** HE staining of tibia **(F)** and quantification analysis of cortical bone thickness **(G)** (bar=100 μm); **(H)** Displacement-load curve and quantification analysis of three-point bending examination. Data are presented as mean ± SEM. Statistic significances are determined by one-way ANOVAs with Dunnett’s multiple comparison tests, with ns: no significance, *P < 0.05, **P < 0.01.

GDD patients are characterized by enhancing bone mass and thickening of cortical bone, which is replicated in *Ano5^KI/KI^* mice. To further investigate the impact of AMPK on abnormal bone phenotype in GDD, *Ano5^KI/KI^* mice were given Compound C by gavage for 4 weeks. μCT was utilized to observe the bone microstructure and showed that inhibiting AMPK could attenuate cortical bone thickness in the middle tibias of *Ano5^KI/KI^* mice, accompanied by declined BMD and BMC (**Figures 7A, B**). BV/TV analysis manifested that disruption of AMPK signaling also rescued the cancellous bone mass of tibias in *Ano5^KI/KI^* mice. Additionally, Compound C treatment resulted in elevated Tb.Th and Tb.N along with declined Tb.Sp in tibias compared with NaCl group (**Figure 7C**). Cortical and trabecular BS/BV, indirectly reflecting osteoclast activity, was both declined in tibia of *Ano5^KI/KI^* mice compared with wildtype group, which was consistent with inhibited osteoclastogenesis caused by *Ano5* mutation (**Supplementary Figure S4A**). Notably, blocking AMPK upregulated the ratio of bone surface to bone volume compared with NaCl treatment group. In femurs, AMPK disruption also exhibited a higher BS/BV, a lower cortical bone mass and a higher trabecular bone phenotype relative to NaCl treatment (**Figure 7D**; **Supplementary Figures S4B–D**). Furthermore, compound C reduced the thickness of the palatal cortical bone of the first molar in mandible, implying blocking AMPK could rescue swell mandible of GDD patients (**Figure 7E**). HE staining of tibia also showed that enhanced thickening of cortical bone in *Ano5^KI/KI^* mice was rescued by AMPK inhibitor (**Figures 7F, G**).

In addition, frequent fracture is one of the most common symptoms for GDD patients. Thus, we directly examined the effect of Compound C on bone mechanical property of tibia by using three-point bending tests (**Figure 7H**). Decreased ultimate load and fracture load were improved by Compound C treatment, accompanied by elevated ultimate stress that are related to cortical thickness. AMPK disruption also improve elastic modulus of *Ano5^KI/KI^* mice, a vital marker of biomechanical properties (**Figure 7H**; **Supplementary Figure S4E**).

**Figure 8:**
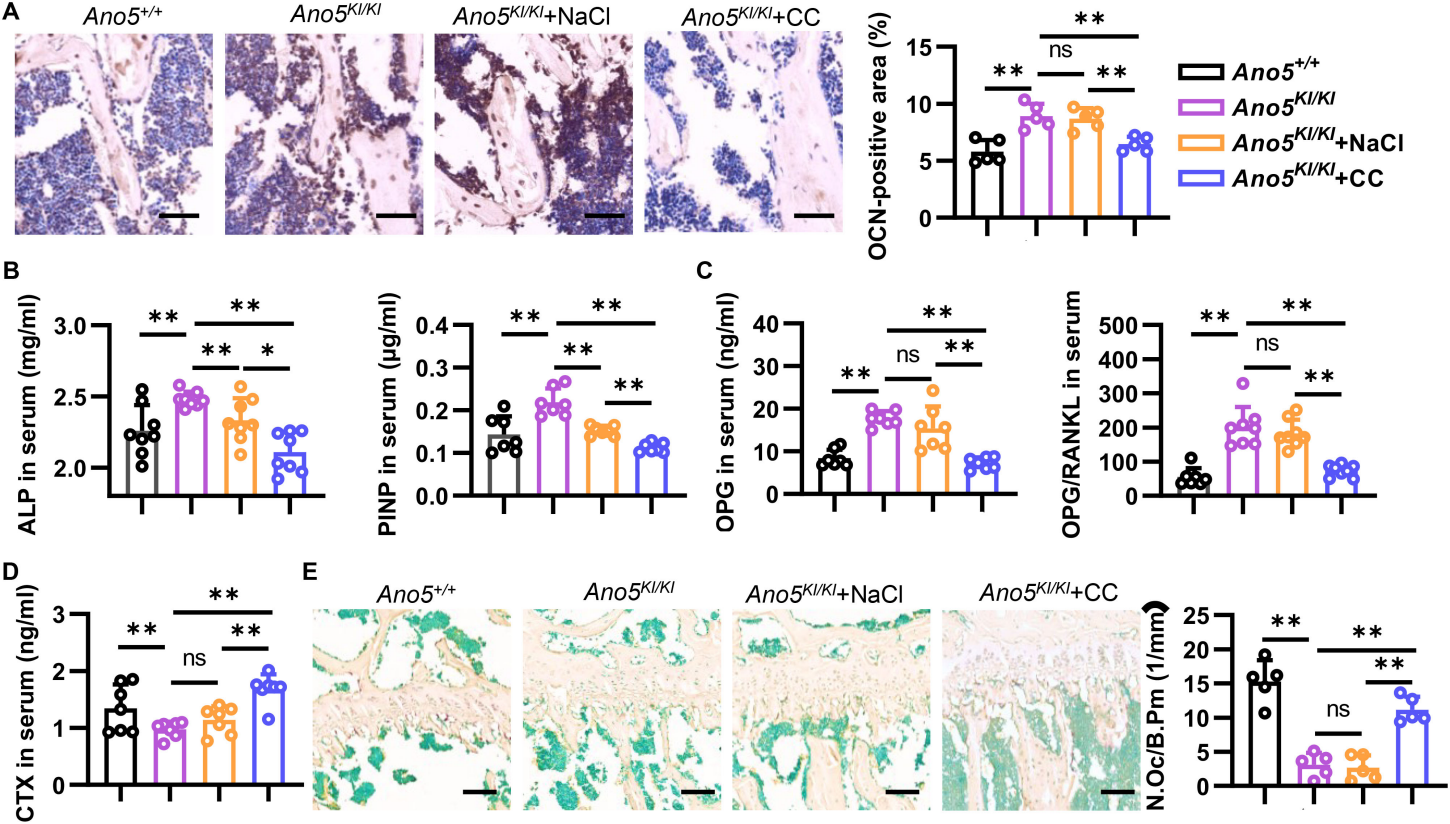
AMPK inhibitor regulates osteogenesis and osteoclastogenesis of GDD *in vivo*. 12-week-old male mice were used to observe bone phenotype. **(A)** Representative photomicrographs and quantitative immuno-positive area analysis of OCN in tibia (bar=100 μm); **(B–D)** ELISA analysis of serum level of ALP, PINP **(B)**, OPG, OPG/RANKL **(C)**, and CTX **(D, E)** Representative images of TRAP staining and quantitative analysis of the osteoclast number (Oc.N)/perimeter of bone (B.Pm) in the germinal center of the tibia (bar=100 μm). Data are presented as mean ± SEM. Statistic significances are determined by one-way ANOVAs with Dunnett’s multiple comparison tests, with ns: no significance, *P < 0.05, **P < 0.01.

As for the immunohistological analysis, the results suggested that the enhanced expression of OCN in the trabecular bone of *Ano5^KI/KI^* mice was rescued by the AMPK inhibitor (**Figure 8A**). Importantly, Compound C significantly reduced the serum ALP level in *Ano5^KI/KI^* mice compared with NaCl group. The elevated P1NP, a classical marker of new bone formation, was also abolished, implying that AMPK inhibitor could mitigate osteogenesis of GDD *in vivo* (**Figure 8B**). Furthermore, Compound C remarkably reduced the serum level of OPG and the ratio of OPG to RANKL in *Ano5^KI/KI^* mice (**Figure 8C**). CTX is released from intact type I collagen by CTSK cleavage and is closely related to the activity of bone resorption. ELISA analysis displayed that declined CTX serum level in *Ano5^KI/KI^* mice was increased by Compound C treatment (**Figure 8D**). Consistently, TRAP staining revealed that AMPK inhibitor obviously promoted the maturation of osteoclasts in *Ano5^KI/KI^* mice (**Figure 8E**). These results further indicate that the effect of the intervention on bone phenotype of GDD by AMPK inhibitor is closely related to decreased osteogenesis and attenuated osteoclastogenesis.

## 4 Discussion

GDD is a rare hereditary skeletal syndrome involved in mandible enlargement and diaphyseal cortical thickening. Clinical treatment for GDD is limited in symptomatic surgery such as fracture fixation and jaw resection. It is urgent to elucidate pathological mechanisms of abnormal bone metabolism caused by ANO5 mutation so as to shed light on treatment by using accurate point mutation animal models. Our previous study demonstrated that *Ano5* deficiency leads to enhanced bone formation and declined bone resorption (9, 12, 28), but the specific mechanism remains to be uncovered.

Glucose metabolism is indispensable for maintaining cell activity in multiple biological processes through the release, transfer, storage, and utilization of energy. Numerous studies have identified fuel requirements and bioenergetic properties of bone under physiological conditions, as well as the dysregulation of energy metabolism associated with bone diseases (29). AMPK serves as a central energy sensor and is essential for maintaining metabolic homeostasis. Our study identified *Ano5* mutation led to a noticeably increased phosphorylation of AMPK in osteoblasts as well as osteoclasts. In addition, not only p.Cys360Tyr but also p.Cys356Tyr mutation, the most common mutation site of GDD, upregulated AMPK activation in 293T cell (**Supplementary Figure S5**), which further confirmed the close correlation between GDD and AMPK signaling.

AMPK activation resulted in enhanced bone mass by promoting the mineralization of osteoblasts and suppressing bone resorption of osteoclast (25). Importantly, AMPK inhibitor had an obvious impact on abrogating osteoblast differentiation and matrix mineralization in *Ano5^KI/KI^* osteoblast validated by decreased OCN expression in tibia and declined serum levels of ALP and PINP. Of note, osteoblasts originating from periosteum play a vital role in bone development, postnatal appositional bone growth, adaptation of bone in response to mechanical loads, and repair of fractures. We found *Ano5* mutation upregulated number of osteoblasts located in the periosteum, which could be rescued by the AMPK inhibitor (**Supplementary Figure S4F**). On the other hand, AMPK activation mediated by Sestrin2 inhibits osteoclastogenesis (30). Consistently, *Ano5^KI/KI^* osteoclast is manifested by elevated phosphorylation of AMPK. While aberrant osteoclast maturation and serum CTX level caused by *Ano5^Cys360Tyr^* mutation were rescued by AMPK inhibitor. Our previous study indicated that the *ANO5* mutation is involved in bone homeostasis through the tight coupling of bone formation and resorption by regulating the ratio of OPG to RANKL (12). AMPK inhibitor also could remarkably downregulate the level of OPG and the ratio of OPG to RANKL in *Ano5^KI/KI^* mice. It is reported that activating AMPK signaling could increase mandibular bone density against aging targeted to maintain osteoblast-osteoclast balance (31). Our research also observed that upregulated mandibular BMD in GDD was attenuated by Compound C. Therefore, inhibiting AMPK signaling plays a positive role in rescuing bone phenotype of GDD characterized by decreased cortical bone thickness, declined BMD, elevated cancellous bone mass, and enhanced biomechanical property of long bone. Of note, the discrepancy between cortical bone and trabecular bone was observed, which also occurred in some pathological conditions, including hyperparathyroidism and SOX4 overexpression. Suppressed formation of trabecular bone might be a compensatory response to the thickening of bone cortex. Importantly, the discrepancy is mainly related with the structural differences between the two types of bone tissues and corresponding specificity of regulatory signaling. Spongy trabecular bone with a mass of osteoblasts and osteoclasts in large surface area is more sensitive to pathways with high expression such as Wnt/β-catenin. However, cortical bone is composed of closely arranged bone units with low cell density and is primarily regulated by mechanical signaling pathways such as Piezo1, which implies mechanical signaling may be a key pathological mechanism of GDD. Additionally, it is considerable that the systemic administration of the Compound C may exert effect on metabolically active tissues, including adipose, liver, and muscle, to indirectly modulate bone metabolism through multiple paracrine or endocrine pathways mediated by key factors, such as adiponectin, fibroblast growth factor 21 (FGF21), insulin-like growth factor binding protein 1 (IGFBP1), IGF1, tumor necrosis factor-alpha (TNF-α), myostatin, irisin, osteopontin (OPN), and so on (32–35). Future studies would address this limitation by using osteoblast-specific or osteoclast-specific AMPK knockout mouse models, injecting AAV9 to enable the targeted deletion of AMPK in bone tissue, or utilizing local delivery systems to specifically modulate AMPK signaling in bone tissue, thereby eliminating the effects of systemic inhibition. Additionally, to analyze changes in key factors secreted by muscles, liver, and adipose tissues following systemic AMPK inhibition would help to clarify the indirect effects on bone metabolism of GDD.

It is well known that AMPK regulates bone homeostasis through promoting autophagy. Our previous study showed that *Ano5* knock-out results in enhanced autophagy in osteoblast, but there is no obvious effect of *Ano5^Cys360Tyr^* mutation on autophagy, which may be attributed to different protein functions relying on the structural domain. Therefore, we focus on whether AMPK-dependent energy metabolism dysregulation is the pivotal mechanism for GDD. During osteoblast differentiation, aerobic glycolysis is a prominent feature by rapid generation of ATP. We also found upregulation of LDH activity and HK2 expression with osteogenic differentiation. It is reported that deletion Notch promotes bone formation attributed to enhanced AMPK-dependent glycolysis. Although mRNA and protein level of LDHA showed slight upregulation, LDH activity was obviously augmented in *Ano5^KI/KI^* osteoblast accompanied with elevated expression of HK2. Besides of energy provision, glycolytic metabolic intermediates are involved in nucleotide anabolism to promote cell proliferation and our study observed inhibiting glycolysis abrogated proliferation activity of *Ano5^KI/KI^* osteoblast. Several intermediates participate in biosynthesis of extracellular matrix collagen through regulating amino acid metabolism, which is consistent with the up-regulation of amino acid metabolites in *Ano5^KI/KI^* osteoblast shown by our previous metabolomics analysis (36). Terminal metabolite lactate that is elevated in *Ano5^KIKI^* osteoblast could promote osteogenic differentiation by regulating histone lactation H3K18la (37). Consistently, glycolysis inhibitor significantly attenuated osteogenesis of *Ano5^KIKI^* mCOB. Furthermore, elevated glycolysis of *Ano5^KIKI^* osteoblast could be mitigated by AMPK inhibitor.

In addition, oxidative phosphorylation is crucial for osteogenic differentiation during early skeletal development (38). *Ano5^Cys360Tyr^* mutation augmented mitochondrial respiration and OXPHOS complex expression in osteoblast. Active mitochondria played a positive role in bone formation through promoting β-catenin signaling that is increased in *Ano5* knock-out osteoblast observed by another study of our group (39). Oligomycin that disturbs oxidative phosphorylation and electron transport chains significantly abrogated ALP activity in *Ano5^KIKI^* osteoblast. *Ano5^Cys360Tyr^* mutation increased PGC1α expression, a key factor of mitochondria biosynthesis, that could promote the expression of osteoblast-related genes (40). AMPK could indirectly up-regulate PGC1α through Sirt1, p38/MAPK, and TFEB signaling. PGC1α knockdown inhibited osteogenic differentiation induced by AMPK under glucose starvation (26). Our study also revealed that AMPK inhibitor play a negative role in PGC1α mRNA and protein level accompanied by decreased mitochondrial respiration in *Ano5^KIKI^* osteoblast. Total ATP generation declined after AMPK inhibitor exposure, which may be due to both abrogated glycolysis and oxidative phosphorylation. Combined with the suppressed effect of AMPK inhibitor on bone formation, it is speculated that elevated glucose energy metabolism mediated by AMPK activation in osteoblast is a pivotal mechanism of enhanced bone formation in GDD.

Similar to osteopetrosis with high bone mass caused by mutations in genes involved in osteoclast differentiation or function, impairment of osteoclastic activation is an important pathological feature of GDD. Osteoclasts are polynucleated cells that are formed by the fusion of precursor cells from the monocyte/macrophage lineage. This process requires numerous energies originating from mitochondrial respiration. However, *Ano5^Cys360Tyr^* mutation damaged mitochondrial structure in osteoclast without apparent alternation in number. Mitochondrial respiration was dramatically inhibited featured by declined ATP generation and OXPHOS complex expression. PGC-1β, another PGC-1 family member, is mainly expressed in osteoclasts to maintain mitochondrial biogenesis and function. Notably, *Ano5^Cys360Tyr^* mutation interfered with PGC1β expression in osteoclasts. PGC1β knockout mice showed increased old bone mass caused by reduced serum CTX and abnormal osteoclast function, which is consistent with enhanced BMD and decreased osteoclastogenesis in GDD. It’s reported that knockdown optic atrophy type 1 (Opa1) that is regulated by PGC1β leads to abnormal cristae morphology in mitochondria (41). *Ano5^KI/KI^* osteoclast also showed aberrant mitochondrial structure, further implying PGC1β dysregulation is closely related to decreased osteoclastogenesis in GDD. H3K27me3 that is suppressed by the interaction between AMPK and EZH2 could directly bind to the promoter of PGC1β to inhibit transcription (42, 43). Conversely, the expression of PGC1β is regulated by CREB and PPARγ, on which AMPK exerts a negative regulatory effect (44, 45). In this study, we observed that the expression of PGC1β was improved by AMPK inhibitor and the molecular mechanism needs to be explored.

The balance between PGC1α and PGC1β exerts a vital impact on bone homeostasis via promoting mitochondrial biogenesis and function to respectively regulate osteoblastogenesis and osteoclastogenesis. Excessive bone formation of osteoblasts caused by the increase of PGC1α and/or insufficient bone resorption of osteoclasts caused by the decrease of PGC1β lead(s) to osteopetrosis (46). Consistently, enhanced PGC1α in osteoblast and declined PGC1β in osteoclast were observed in GDD. Inhibiting AMPK *in vitro* could respectively reverse the effects of PGC1α and PGC1β on mitochondrial respiratory function in *Ano5^Cys360Tyr^* osteoblasts and osteoclasts. Our study preliminarily clarifies that the mitochondrial respiratory imbalance mediated by PGC1α/PGC1β that is attributed to excessive activation of AMPK is closely related to the enhanced bone mass of GDD. Several researchers point out that HIF1α could regulate AMPK signaling to promote MSC proliferation, migration, and osteogenic differentiation (47). HIF1α, the key regulator of energy metabolism and glycolysis, plays a positive role in bone formation to improve cortical bone phenotype (48), which is similar to enhanced cortical thickness of GDD. Although the mRNA expression was comparable between *Ano5^KI/KI^* mCOB and wildtype group, *Ano5^Cys360Tyr^* mutation obviously increased the protein level of HIF1α in mCOB at day 0, indicating enhanced HIF1α expression may be the key mechanism by which *Ano5* mutation activates AMPK signaling in osteoblasts (**Supplementary Figures S5A, B**). Additionally, HIF1α is the vital positive regulatory factor for osteoclast differentiation (49). we found *Ano5* mutation reduced *Hif1α* expression in BMMs without osteoclast differentiation. Because of the instability of protein, the HIF1α protein expression under 0.2% O_2_ condition in osteoblasts and osteoclasts is needed to be examined to further confirm the relation between HIF1α and abnormal bone phenotype of GDD. AMPK can be activated under high levels of cellular ROS and maintains redox homeostasis (50). However, ANO5 mutation reduced the cellular ROS level in both osteoclast and osteoblast. Ca^2+^/CAMKK2 is one of the classical signaling to activate AMPK phosphorylation (51), but our previous study found that *Ano5^Cys360Tyr^* mutation led to a decrease in intracellular free calcium in osteoblasts and osteoclasts (12). Another study also observed that *Ano5* knockdown suppressed calcium oscillation in skeletal cells (9). *Ano5^Cys360Tyr^* mutation decreased the protein level of glucose transporter type 1 (GLUT1) responsible to glucose uptake (**Supplementary Figure S6A**). Glucose uptake probe-red detection also exhibited that *Ano5^Cys360Tyr^* mutation inhibited glucose uptake in osteoblasts (**Supplementary Figure S6B**), further implying AMPK could be activated by starvation (52).

**Figure 9:**
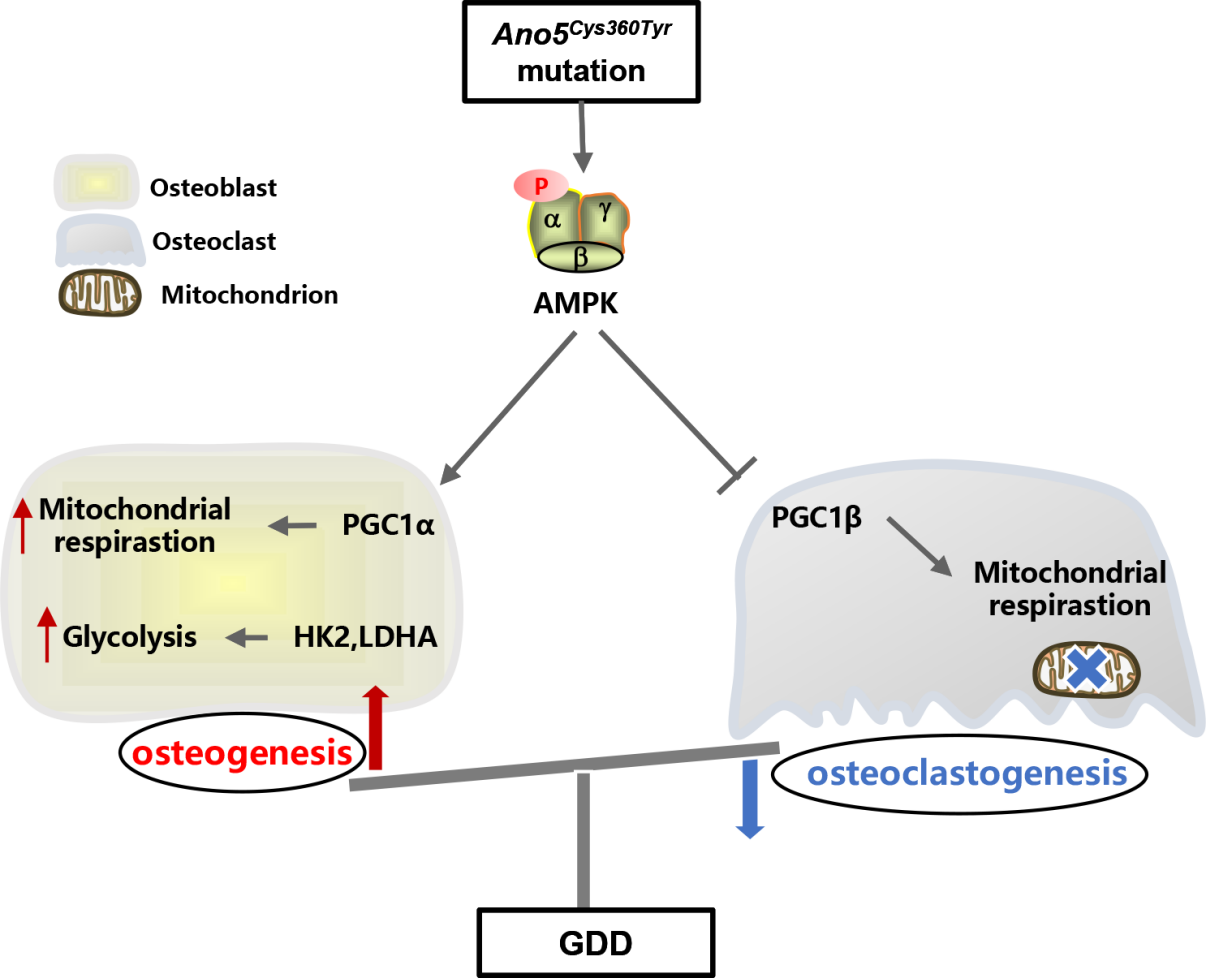
Schematic illustration of glucose metabolic distemperedness mediated by AMPK activation in GDD. Excessive AMPK activation caused by *ANO5^Cys360Tyr^* mutation stimulated glycolysis in osteoblast and disturbed the mitochondrial homeostasis between osteoblast and osteoclast by enhancing PGC1α and inhibiting PGC1β expression respectively, to augment bone formation and suppress osteoclastogenesis.

In conclusion, we revealed that *Ano5^Cys360Tyr^* mutation disturbed the balance of glucose metabolism between osteoblast and osteoclast mediated by excessive AMPK activation (**Figure 9**). Blocking AMPK could attenuate osteogenesis and augment osteoclastogenesis to rescue enhanced bone mass of GDD *in vitro* and *in vivo*, which provides a promising target for the future therapies against GDD.

## Data Availability

The original contributions presented in the study are included in the article/**Supplementary Material**. Further inquiries can be directed to the corresponding authors.
