# Supplementary material for: Anoctamin 5 mutation leads to abnormal bone homeostasis of GDD by regulating AMPK-dependent glucose metabolism
Source: Front Endocrinol (Lausanne). 2026 Feb 4;17:1703491. doi: 10.3389/fendo.2026.1703491 (PMC12913092; doi:10.3389/fendo.2026.1703491)
Supplement: Supplementary file 1 [file DataSheet1.docx]

Supplementary Material

# Supplementary Figures and Tables

## Supplementary Figures


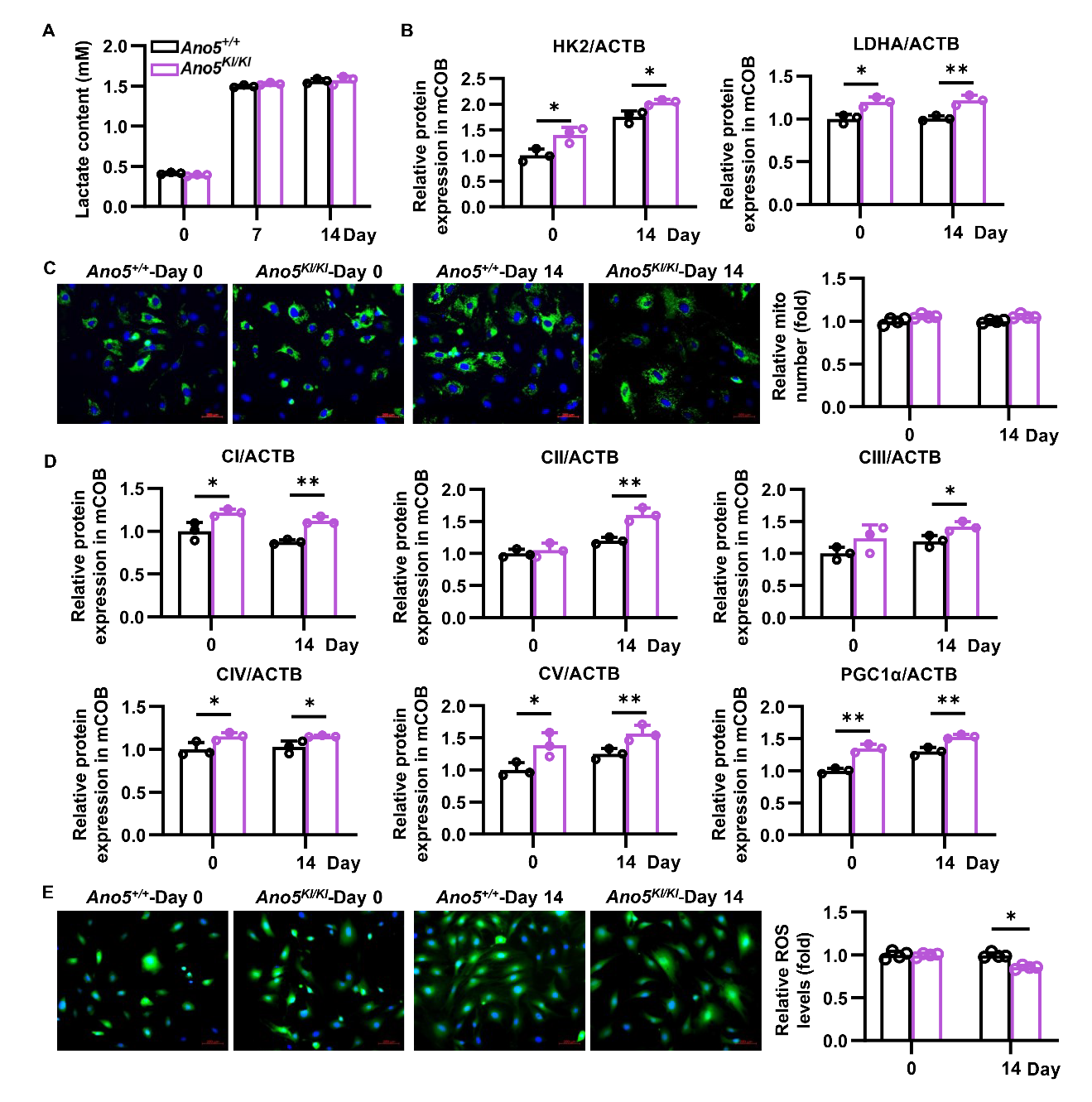
 **Supplementary Figure 1. *Ano5^Cys360Tyr^* mutation affects energy metabolism in osteoblast.** (A) lactate level in supernatant of mCOB at days 0 and 14 (*n*=4); (B) Relative quantification of HK2 and LDHA in mCOB in Figure 1C (*n*=3); (C) Immunofluoresence images (green) and relative quantitative analysis of mitochondrial number (*n*=4); (D) Relative quantification of OXPHOS complex and PGC1α in mCOB in Figure 1F and Figure 1G (*n*=3); (E) Immunofluoresence images (green) and relative quantitative analysis of ROS level (*n*=4). Data are presented as mean ± SEM. Statistic significance are determined by one-way ANOVAs with Dunnett's multiple comparison tests, with *P<0.05.


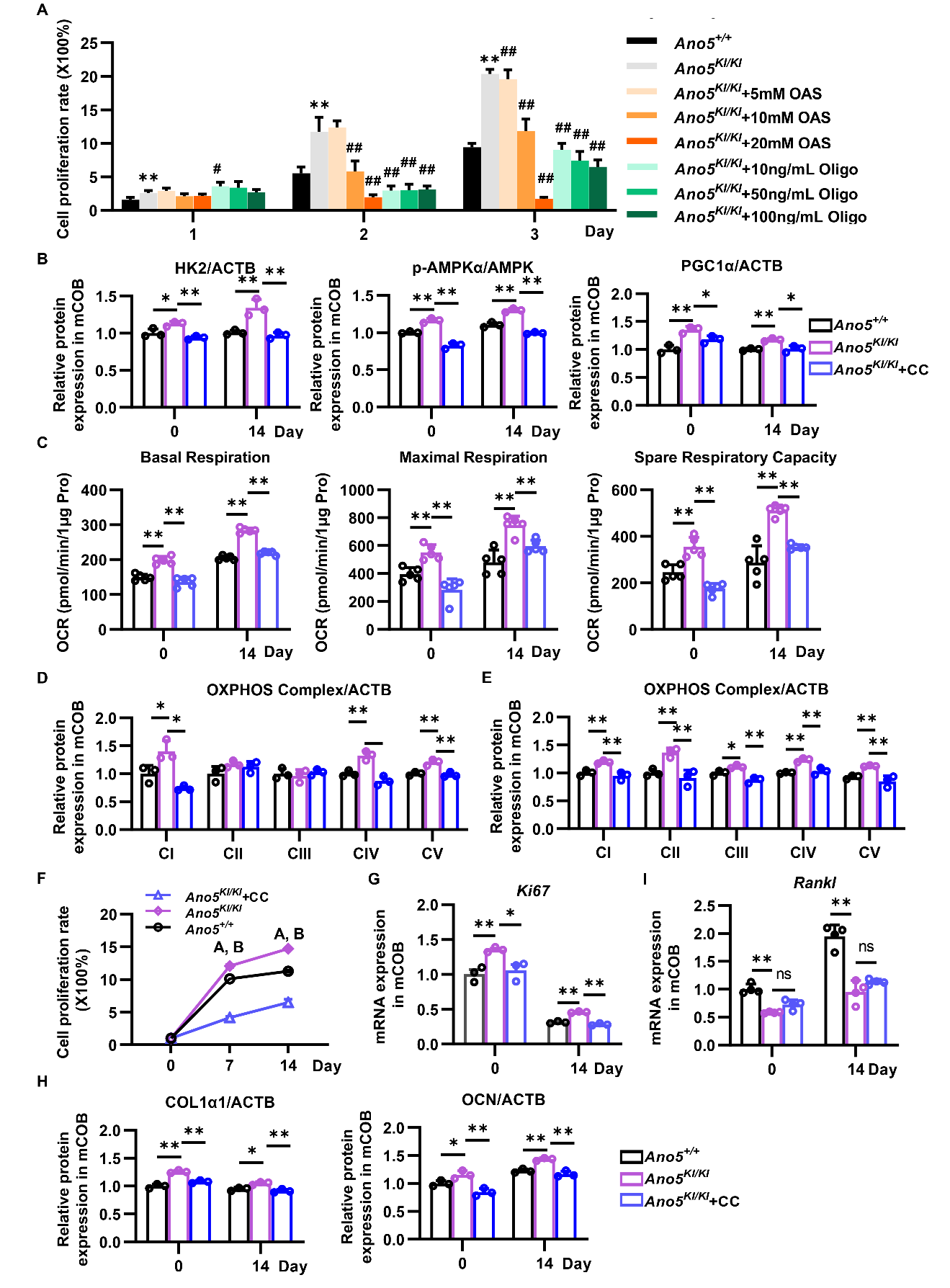
**Supplementary Figure 2: AMPK-dependent glucose metabolism leads to enhanced osteogenesis of *Ano5^KI/KI^* mCOB attributed to.** (A) cell proliferation analysis of *Ano5^KI/KI^* mCOB exposure to glycolysis inhibitor OAS and mitochondrial respiration blocker Oligo at days 1, 2, and 3 without osteogenic induction by CCK8 detection (***P*<0.01 representing *Ano5^KI/KI^* *vs* *Ano5^+/+^*, *^#^P*<0.05 and *^##^P*<0.01 representing *Ano5^KI/KI^*+ stimulation *vs* *Ano5^KI/KI^*); (B) Relative quantification of HK2, p-AMPK/AMPK, and PGC1α in mCOB (*n*=3); (C) Quantitative analysis of Seahorse XF mito stress in Figure 3I, including basal respiration, maximal respiration, and spare respiration (*n*=5); (D-E) Relative quantification of OXPHOS complex in mCOB at days 0 (D) and days 14 (E) of osteogenic induction in Figure 3H; (F) CCK8 detection of mCOB at days 7 and 14 with osteogenic induction (*^A^P*<0.01 representing *Ano5^KI/KI^* *vs* *Ano5^+/+^* and *^B^P*<0.01 representing *Ano5^KI/KI^*+Compound C *vs* *Ano5^KI/KI^*); (G) qRT-PCR analysis of *Ki67* of mCOB at days 0 and 14 (*n*=3); (H) Relative quantification of COL1α1 and OCN in mCOB in Figure 4F (*n*=3); (I) qRT-PCR analysis of *Rankl* in mCOB at days 0 and 14 (*n*=3). Data are presented as mean ± SEM. Statistic significance are determined by one-way ANOVAs with Dunnett's multiple comparison tests, with *P<0.05.


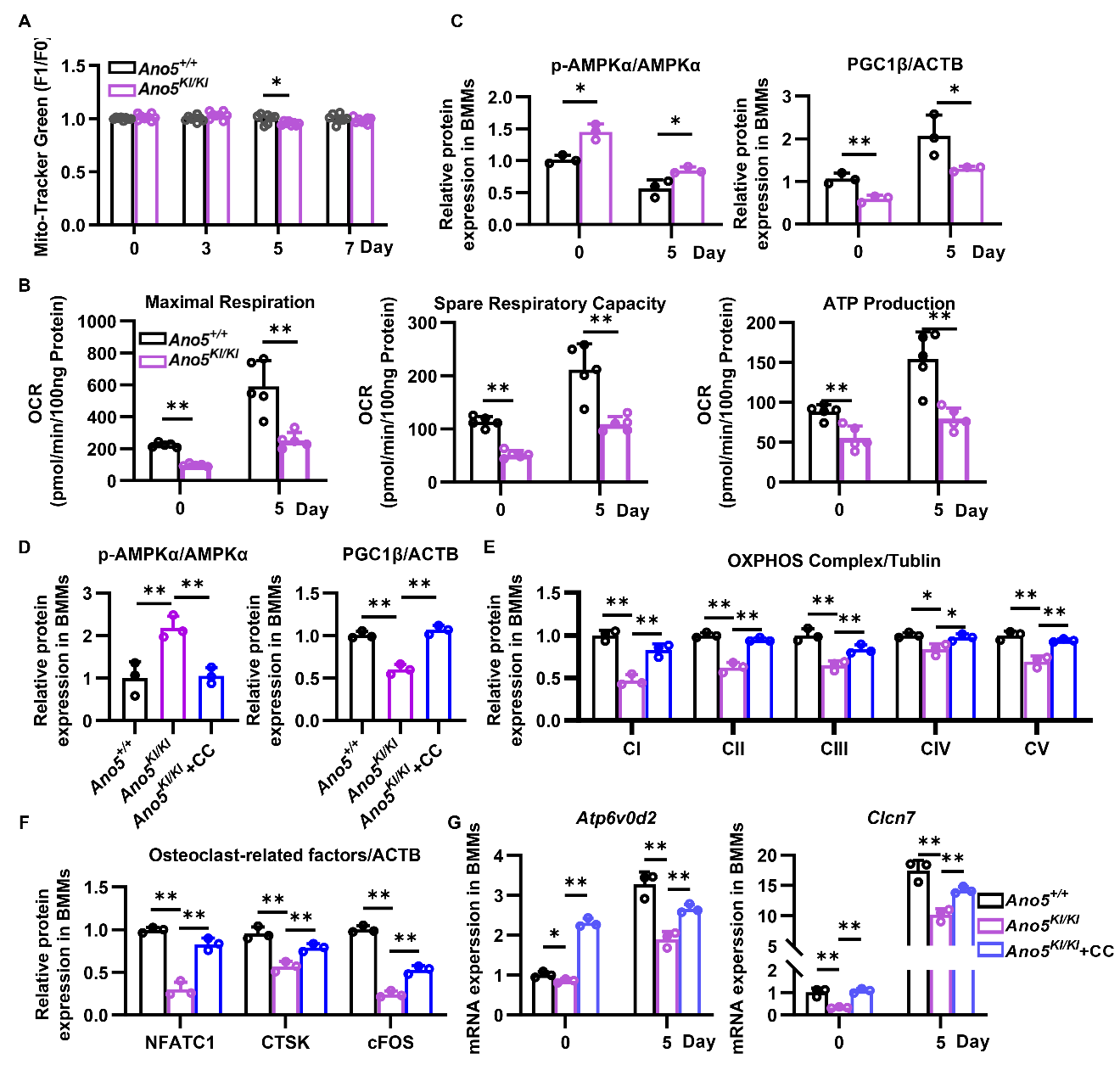
 **Supplementary Figure 3. *Ano5^Cys360Tyr^* mutation disturbs mitochondrial of osteoclast.** (A) Mitochondrial number detection of *Ano5^+/+^* and *Ano5^KI/KI^* osteoclast by mito-track green staining at different stages of osteoclast differentiation (*n*=6); (B) Quantitative analysis of Seahorse XF mito stress in Figure 5E, including maximal respiration, spare respiration, and ATP production ability (*n*=5); (C) Relative quantification of p-AMPK/AMPK and PGC1β in BMMs in Figure 5F (*n*=3); (D) Relative quantification of p-AMPK/AMPK and PGC1β in BMMs with Compound C treatment at days 5 of osteoclast differentiation in Figure 5G (*n*=3); (E) Relative quantification of OXPHOS complex in BMMs with Compound C treatment at days 5 of osteoclast differentiation in Figure 5H (*n*=3); (F) Relative quantification of NFATC1, CTSK, and cFOS in mature osteoclasts in Figure 6B (*n*=3); (G) qRT-PCR analysis of *Atp6v0d2* and *Clcn7* (*n*=3). Data are presented as mean ± SEM. Statistic significance are determined by t-tests (A, B) or one-way ANOVAs with Dunnett's multiple comparison tests (C), with **P<0.05*.


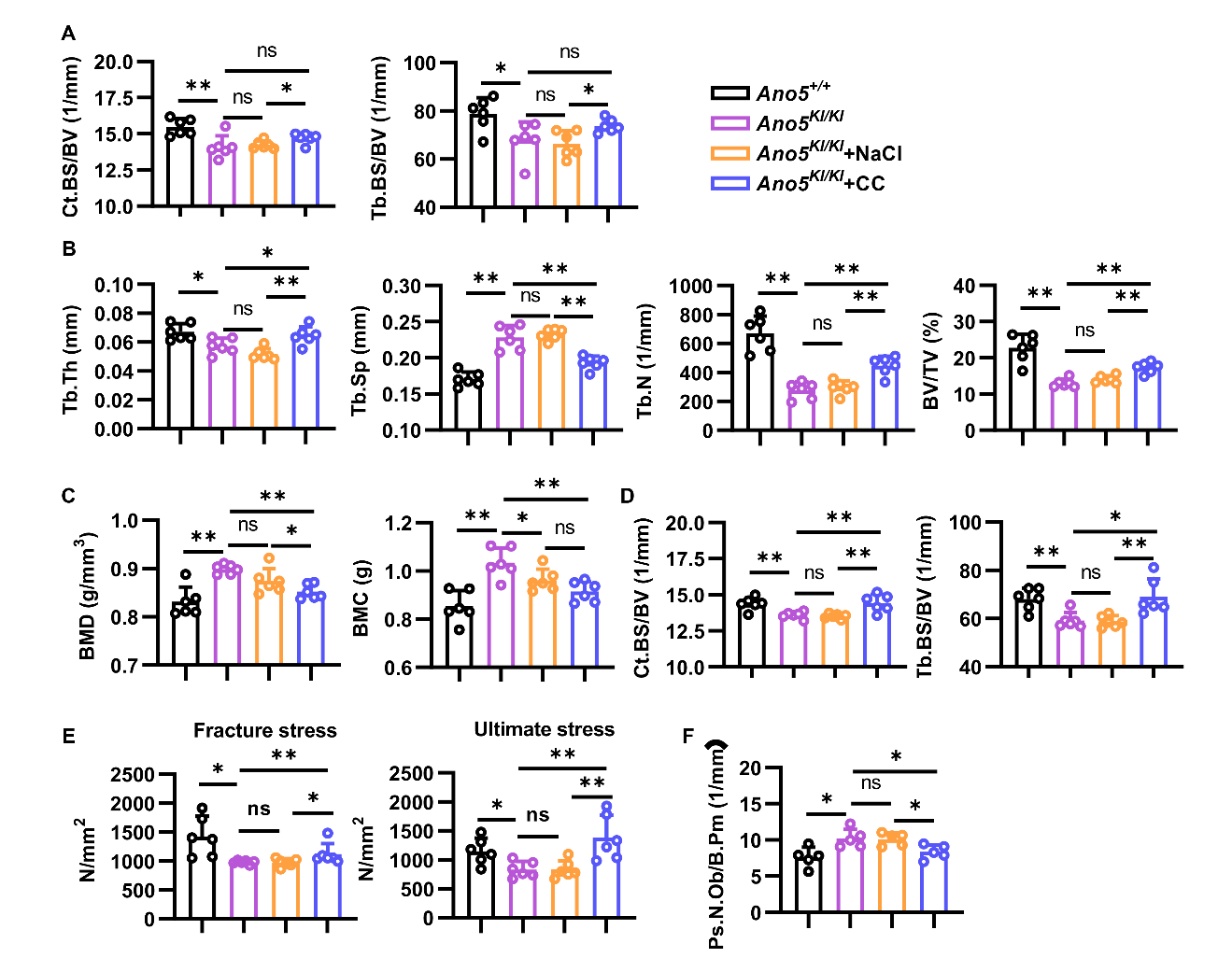

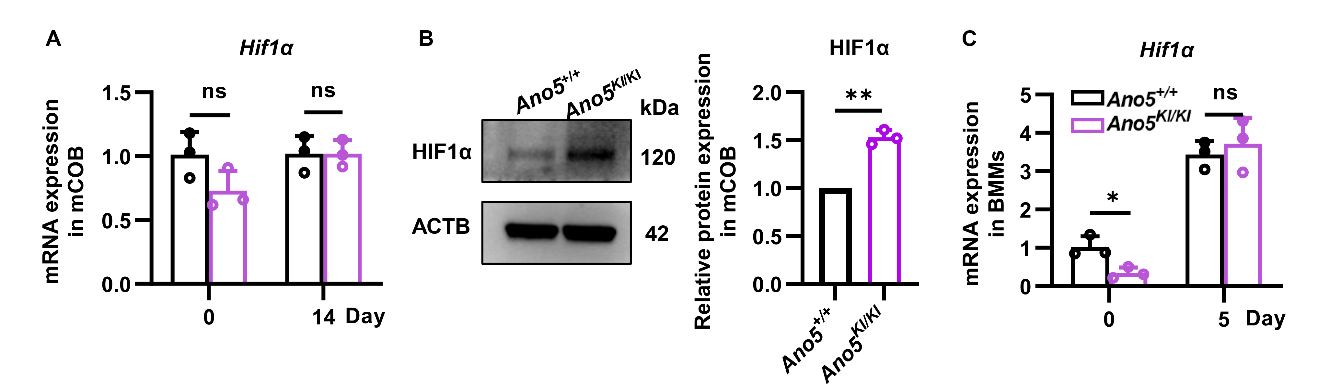
**Supplementary Figure 4. AMPK inhibitor Compound C rescues bone phenotype of GDD.** (A) Analysis of cortical BS/BV (Ct.BS/BV) and trabecular BS/BV (Tb.BS/BV) of tibia; (B-C) Quantification analysis of μCT analysis of trabecula bone (B) and cortical bone (C) and of femur (*n*=6); (D) Analysis of Ct.BS/BV and tTb.BS/BV of femur; (E) Quantification analysis of fracture stress and ultimate stress by three-point bending experiment (*n*=6); (F) Quantification analysis of number of periosteal osteoblasts per bone surface (Ps.N.Ob/BS) of tibia (*n*=5). Statistic significance are determined by one-way ANOVAs with Dunnett's multiple comparison tests, with ns: no significance, **P*<0.05, ***P*<0.01.

**Supplementary Figure 5. *Ano5^Cys360Tyr^* mutation regulates HIF1α expression.** (A) qRT-PCR analysis of *Hif1α* in mCOB at days 0 and 14 of osteogenic differentiation (*n*=3). (B) Immunoblotting analysis of HIF1α in mCOB at day 0 (*n*=3). (C) qRT-PCR analysis of *Hif1α* in BMMs at days 0 and 5 of osteoclast differentiation (*n*=3). Data are presented as mean ± SEM. Statistic significance are determined by t-tests, with ns: no significance, **P*<0.05, ***P*<0.01.


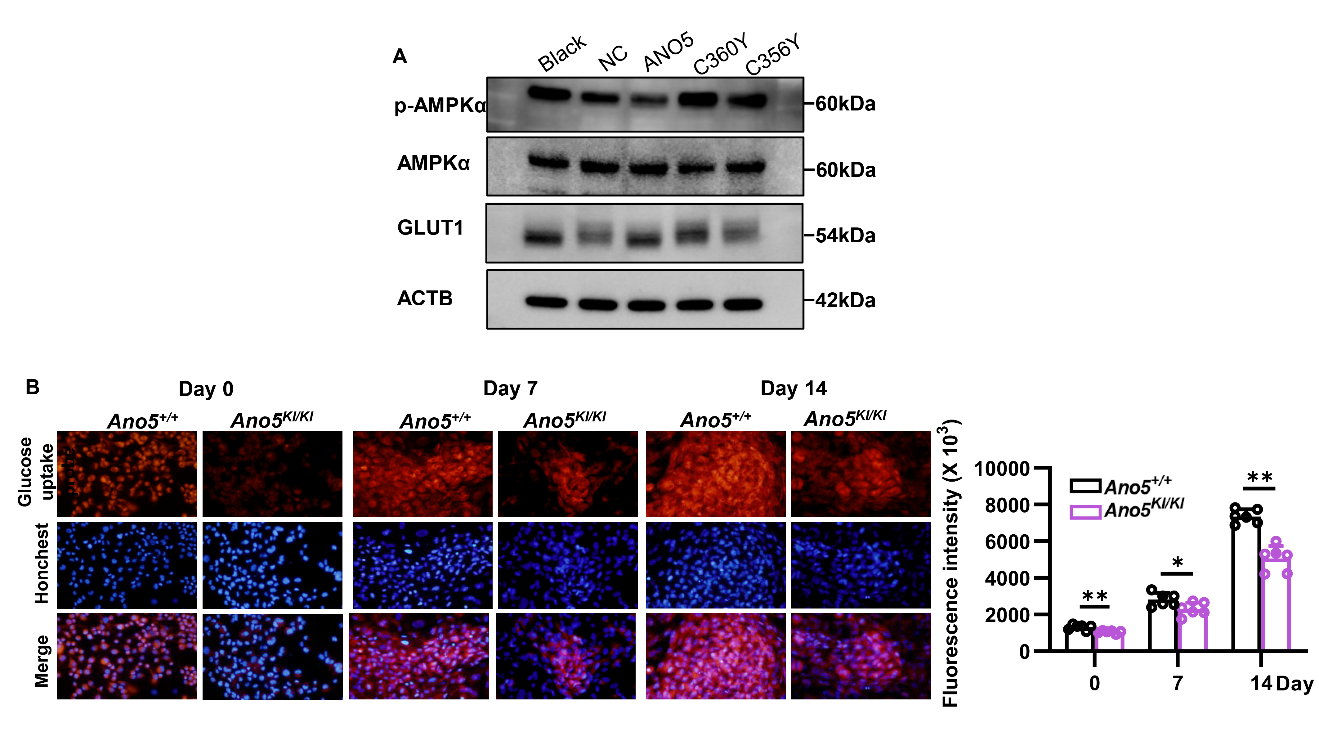
 **Supplementary Figure 6. ANO5 mutation inhibits glucose uptake.** (A) Immunoblotting analysis of p-AMPKα/AMPKα, GLUT1, and ACTB in HEK293T of black, NC, ANO5 overexpression, ANO5^Cys360Tyr^ overexpression, and ANO5^Cys356Tyr^ overexpression. (B) Glucose uptake probe-Red detection of *Ano5^+/+^* and *Ano5^KI/KI^* mCOB at days 0, 7, and 14 of osteogenic differentiation. Statistic significance are determined by t-tests, **P*<0.05, ***P*<0.01.

## Supplementary Tables

**Supplementary Table 1. Primer sequences of genes in qRT-PCR assay**

| Gene | Forward Primer | Reversed Primer |
| --- | --- | --- |
| *Actb* | GTGACGTTGACATCCGTAAAGA | GCCGGACTCATCGTACTCC |
| *Ocn* | GAACAGACAAGTCCCACACAGC | TCAGCAGAGTGAGCAGAAAGAT |
| *ColIαI* | TTCTCCTGGTAAAGATGGTGC | GGACCAGCATCACCTTTAACA |
| *Runx2* | GGCAAGATGAGCGACGTGAG | ATCTGACTCTGTCCTTGTGG |
| *Opg* | GAGCAAACCTTCCAGCTGC | TGCTCTGTGGTGAGGTTCG |
| *Nfatc1* | TGCTCCTCCTCCTGCTGCTC | CGTCTTCCACCTCCACGTCG |
| *Ctsk* | CTTCCAATACGTGCAGCAGA | TCTTCAGGGCTTTCTCGTTC |
| *cFos* | CAAGCGGAGACAGATCAACTTG | TTTCCTTCTCTTTCAGCAGATTGG |
| *Clcn7* | GCTGGTGGAGTTCCTGAAGA | CAGGACCAGGAAGCAGGTAG |
| *Atp6v0d2* | AAGCCTTTGTTTGACGCTGT | TTCGATGCCTCTGTGAGATG |
| *Dc-stamp* | AAAACCCTTGGGCTGTTCTT | AATCATGGACGACTCCTTGG |
| *Mmp9* | CTTCCCTCTGAATAAAGACGACA | CTGTAATGGGCTTCCTCTATGATT |
| *Trap* | CAGCTCCCTAGAAGATGGATTCAT | GTCAGGAGTGGGAGCCATATG |
| *Pgc1α* | AACAATGAGCCTGCGAAC | CCTCGTTGTCAGTGGTCA |
| *Hk2* | TGATCGCCTGCTTATTCACGG | AACCGCCTAGAAATCTCCAGA |
| *Ldha* | GGATGAGCTTGCCCTTGTTGA | GACCAGCTTGGAGTTCGCAGTTA |
| *Atp5b* | CGTGAGGGCAATGATTTATACCAT | TCCTGGTCTCTGAAGTATTCAGCAA |
| *Sdhb* | AATTTGCCATTTACCGATGGGA | AGCATCCAACACCATAGGTCC |
| *Cycs* | TCTCCATGCCTCTAACACTCG | CCAGGTCAACAGACGTGTCAG |
| *Cox4* | TACTTCGGTGTGCCTTCGA | TGACATGGGCCACATCAG |
| *Ki67* | GCCTCCTAATACACCACTGAA | GCCGTTCCTTGATGATTGTC |

**Supplementary Table 2. Primary and secondary antibodies in western blot and immunofluorescence assay**

| Antibody | Catalog | Host species | Dilution | Application |
| --- | --- | --- | --- | --- |
| Phospho-AMPKα mAb | CST #2535 | Rabbit | 1:1000 | WB |
| AMPKα mAb | CST #5831 | Rabbit | 1:1000 | WB |
| Total OXPHOS Antibody | Abcam, ab110413 | Rabbit | 1:1000 | WB |
| GLUT1 mAb | Abcam, ab115730 | Rabbit | 1:10000 | WB |
| HK2 pAb | Proteintech, 22029 | Rabbit | 1:10000  1:400 | WB  IF |
| LDHA pAb | Proteintech, 19987 | Rabbit | 1:5000  1:200 | WB  IF |
| PGC1α mAb | Proteintech, 66369 | Mouse | 1:5000 | WB |
| PGC1β mAb | Abcam, ab176328 | Rabbit | 1:5000 | WB |
| OCN pAb | Affinity, DF12303 | Rabbit | 1:1000 | WB |
|  |  |  | 1:100 | IHC |
| COL1α1 mAb | Abclonal, A24112 | Rabbit | 1:1000 | WB |
| NFATC1 mAb | Santa Cruz, sc7294 | Rabbit | 1:200 | WB |
| CTSK pAb | Abcam, ab19027 | Rabbit | 1:1000 | WB |
| cFOS mAb | CST #2250T | Rabbit | 1:1000 | WB |
| HIF1α mAb | CST #36169 | Rabbit | 1:1000 | WB |
| alpha Tubulin mAb | Abcam, ab7291 | Mouse | 1:1000 | WB |
| Actin mAb | Abclonal, AC026 | Rabbit | 1:100000 | WB |
| ABflo® 594-conjugated Goat anti-Rabbit IgG (H+L) | ABclonal, AS039 |  | 1:200 | IF |
| Goat anti-Rabbit IgG (H+L) Secondary Antibody, HRP | Invitrogen, 31460 |  | 1:5000 | WB |
| Goat anti-Mouse IgG (H+L) Secondary Antibody, HRP | Invitrogen, 31430 |  | 1:5000 | WB |
